# An acytokinetic cell division creates PIP2-enriched membrane asymmetries leading to slit diaphragm assembly in *Drosophila* nephrocytes

**DOI:** 10.1242/dev.201708

**Published:** 2023-09-18

**Authors:** Marta Carrasco-Rando, Joaquim Culi, Sonsoles Campuzano, Mar Ruiz-Gómez

**Affiliations:** Centro de Biología Molecular Severo Ochoa, CSIC and UAM, Nicolás Cabrera 1, Cantoblanco 28049, Madrid, Spain

**Keywords:** Acytokinetic cell division, Membrane symmetry breaking, Slit diaphragm, Nephrocyte, *Drosophila*

## Abstract

Vertebrate podocytes and *Drosophila* nephrocytes display slit diaphragms, specialised cell junctions that are essential for the execution of the basic excretory function of ultrafiltration. To elucidate the mechanisms of slit diaphragm assembly we have studied their formation in *Drosophila* embryonic garland nephrocytes. These cells of mesenchymal origin lack overt apical-basal polarity. We find that their initial membrane symmetry is broken by an acytokinetic cell division that generates PIP2-enriched domains at their equator. The PIP2-enriched equatorial cortex becomes a favourable domain for hosting slit diaphragm proteins and the assembly of the first slit diaphragms. Indeed, when this division is either prevented or forced to complete cytokinesis, the formation of diaphragms is delayed to larval stages. Furthermore, although apical polarity determinants also accumulate at the equatorial cortex, they do not appear to participate in the recruitment of slit diaphragm proteins. The mechanisms we describe allow the acquisition of functional nephrocytes in embryos, which may confer on them a biological advantage similar to the formation of the first vertebrate kidney, the pronephros.

## Teaser

**Summary:** An acytokinetic cell division breaks the initial membrane symmetry in embryonic garland nephrocytes creating PIP2-enriched membrane microdomains suitable for slit diaphragm assembly.

## INTRODUCTION

*Drosophila* nephrocytes, the excretory cells of the fly engaged in haemolymph ultrafiltration, possess filtration diaphragms that are homologous to the podocyte slit diaphragm, a key component of the glomerular filtration barrier (Weavers et al., 2009; Zhuang et al., 2009). Both kinds of diaphragms are modified cell junctions that function as molecular filters during the process of ultrafiltration. Their main constituents, nephrin (Sticks and stones, Sns in *Drosophila*) and NEPH1 (also known as KIRREL1; Dumbfounded, Duf or Kirre in *Drosophila*), are transmembrane adhesion molecules of the immunoglobulin superfamily, the extracellular domains of which contribute to the formation of the sieves. The short cytoplasmic tails associate with other members of the slit diaphragm interactome, such as ZO1 (TJP1l; Polychaetoid, Pyd in *Drosophila*; Weavers et al., 2009), providing anchoring to the cytoskeleton and regulating the stability of the structure as well as many aspects of the podocyte/nephrocyte biology, including their cytoarchitecture and physiology (Benzing, 2004; Grahammer et al., 2013). Insults affecting the glomerular filtration barrier lead to the pathological destabilisation of slit diaphragms, with the mobilisation of their constituents to ectopic occludens-like junctions. Conversely, during development and processes of slit diaphragm repair after damage, the opposite reorganisation of structures takes place, with the transition of slit diaphragm components from junctional complexes to slit diaphragms (Ichimura et al., 2016; Kriz et al., 2013). Similar junctional remodelling has been described in *Drosophila* larval nephrocytes (Carrasco-Rando et al., 2019). Several studies have focused on the transition from junctional complexes to slit diaphragms during podocyte development. Thus, at early developmental stages, the cells of the visceral glomerular epithelium that face the prospective Bowman space (immature podocytes) are joined by occluding-like junctions at their apices. As development proceeds, these cell junctions migrate along the lateral membrane towards the basal side, resting on the basement membrane. Once there, they start to disintegrate, and are replaced by slit diaphragm structures, coinciding with the elaboration and early interdigitation of developing foot processes (Ichimura et al., 2016; Reeves et al., 1978). During this transition, both ZO1, a cell junction and slit diaphragm protein, and members of the PAR3/PAR6/aPKC polarity complex, which are initially localised at the apical junctional complexes, migrate basally and accumulate at the position of the forming slit diaphragms, where the slit diaphragm components nephrin and podocin also localise (Huber et al., 2009; Schnabel et al., 1990). These findings, together with the reported requirement of aPKCλ for the correct distribution of slit diaphragm proteins and slit diaphragm maintenance in podocytes, led to the proposal that aPKCλ function could be instrumental for the differentiation of foot processes and the targeting of slit diaphragm components to their final location (Hirose et al., 2009; Huber et al., 2009). Recently it has been described that *Drosophila* larval nephrocytes accumulate apical-basal polarity proteins, and that aPKC also plays a role in the maintenance of slit diaphragms, as its attenuation in nephrocytes induced both endocytosis and slit diaphragm defects (Heiden et al., 2021; Koehler et al., 2022; Mysh and Poulton, 2021). Slit diaphragm junctions are unique in including adhesion molecules of the immunoglobulin superfamily known as IRM proteins (Irre Cell Recognition Module; Fischbach et al., 2009), comprising nephrin, NEPH1 and their orthologues, *Drosophila* Sns and Duf among them, which in vertebrates have been described to partition into lipid raft membrane fractions enriched in PIP2 (Arif et al., 2011, 2014; Huber, 2003; Schwarz et al., 2001; Simons et al., 2001). However, the question of how the slit diaphragm components are directed towards their final membrane destination remains unanswered.

*Drosophila* has two kinds of nephrocytes: garland and pericardial, which derive from the posterior secondary head mesoderm and the dorsal trunk mesoderm, respectively (de Velasco et al., 2006; Rugendorff et al., 1994). Interestingly, although both types of nephrocytes emerge during embryonic stage 11 (Carmena et al., 1995; Weavers et al., 2009), garland nephrocytes assemble slit diaphragms during embryogenesis, whereas this process is delayed until post-embryonic development in pericardial nephrocytes (Weavers et al., 2009). Another distinguishing feature of garland nephrocytes is that they are binucleated cells (Weavers et al., 2009; Zhuang et al., 2009).

Here, we have studied how slit diaphragms are formed in garland nephrocytes. These cells are of mesenchymal origin, unlike podocytes and, accordingly, lack a defined apical-basal polarity and show very weak cell-cell adhesion. This poses the interesting question of how symmetry is broken in garland nephrocytes to generate distinctive membrane subdomains in which slit diaphragm proteins will be preferentially housed. We find that the emergence of slit diaphragms coincides with the moment when garland nephrocytes become binucleated as result of an acytokinetic cell division (embryonic stage 14). Slit diaphragm proteins mobilise from tricellular contacts, where they reside in mononucleated garland nephrocytes, towards the equatorial cortex when nephrocytes become binucleated, where they are distributed, forming a ring between the sibling nuclei. The equatorial membrane has typical features of a cleavage furrow membrane, such as PIP2 accumulation and the association of Rho1 and the septin protein Peanut, and differs from that of the rest of the nephrocyte. Accumulation of slit diaphragm components is followed by the assembly of the first slit diaphragms close to the equatorial cell cortex, which seal the entrance to membrane invaginations that ingress between the nuclei. At embryonic stage 15 the building of new slit diaphragms continues, such that at the end of embryogenesis they cover the entire exposed surface of the nephrocyte. These observations and additional data obtained from analysis of loss- and gain-of-function mutations for genes affecting cell division, cell polarity and slit diaphragm assembly are consistent with the interpretation that the acytokinetic cell division in garland nephrocytes breaks their typical mesenchymal membrane symmetry, by generating and maintaining PIP2-enriched membrane domains. This creates a favourable environment for the recruitment of slit diaphragm proteins that leads to the nucleation and assembly of slit diaphragms. Furthermore, we show that, although in nephrocytes aPKC and the slit diaphragm proteins preferentially accumulate in PIP2-enriched membrane domains, they do not depend on each other for their targeting to these microdomains, suggesting that PIP2 and not aPKC is the main determinant of mobilisation of slit diaphragm proteins to their final destination.

## RESULTS

### An incomplete cytokinesis induces the accumulation of slit diaphragm proteins at the equatorial cortex in stage 14 garland nephrocytes

**Fig. 1.**
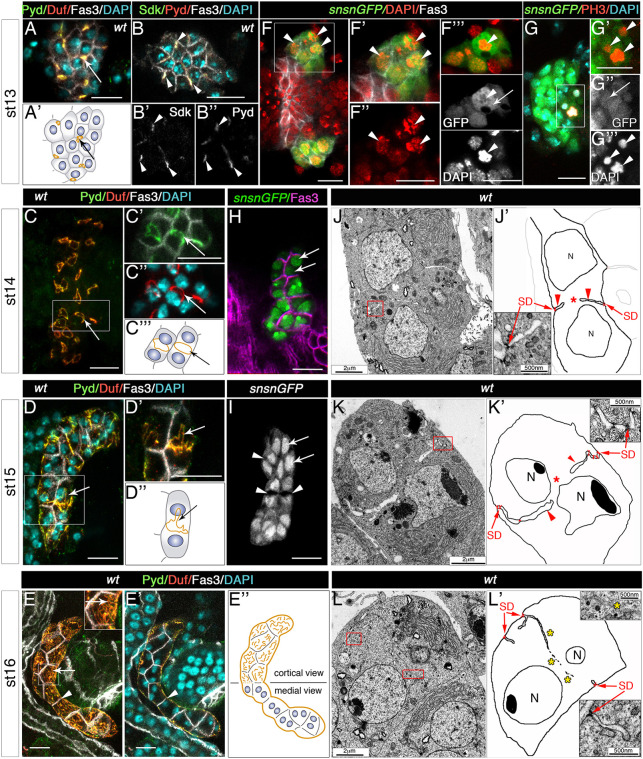
**Genesis of nephrocyte slit diaphragms during embryogenesis.** (A-E″) Confocal images and schematics of wild-type garland nephrocytes at developmental stages 13 to 16, stained with the indicated antibodies. *Z*-projections of stacks are shown except in B-B″, E′ (single planes). (B-B″) Sdk and Pyd colocalise in tricellular contacts [arrowheads, *n*=32 nephrocytes (N)/4 strings (S)]. Arrows in A,A′,C-E point to the accumulation of Duf and Pyd in tricellular contacts (A,A′, *n*=87N/5S) in a ring located at the equatorial cortex (C-C‴, *n*=124N/9S), which becomes distorted in D-D″ (*n*=137N/12S) and in a ruffled pattern on the nephrocyte outer surface (E, *n*=153N/8S). Arrowheads in E,E′ point to Fasciclin 3 (Fas3) accumulation in contact membranes between adjacent nephrocytes. (F-I) Single sections or *z*-projection (G) of *sns-GCN-nGFP* nephrocytes showing the presence of mitotic figures at stage 13 (arrowheads, F-F‴, *n*=32N/7S, DAPI staining; G-G‴, *n*=9N/6S, anti-PH3 staining) in cells that also show cytoplasmic distribution of nGFP (F‴ and G″, arrows); note the nuclear confinement of GFP in binucleated nephrocytes (arrows) at stages 14 (H, *n*=99N/7S) and 15 (I, *n*=71N/4S). White squares denote the regions magnified in adjacent panels; F-F″ and F‴ display the same cluster of nephrocytes at two different focal planes. Arrowheads in I show lumina of membrane invaginations. (J-L′) TEM micrographs and schematics of medial sections of stage 14 (J, *n*=12N), 15 (K, *n*=15N) and 16 (L, *n*=10N) wild-type garland nephrocytes. Red squares indicate enlarged regions in nearby panels, arrows point to slit diaphragms (SD), arrowheads to channels formed by membrane ingressions between sibling nuclei (N) and asterisks to cytoplasmic continuity (J′ and K′) or to membrane fragments (L′) located between the nuclei. Scale bars: 10 µm (A-I); 2 µm (J,K,L); 500 nm (J′,K′,L′).

To study the emergence of slit diaphragms in garland nephrocytes, we analysed the cellular distribution of Duf and Pyd, two of their main constituents, during embryogenesis. At early stage 13, when mononucleated nephrocytes are arranged in a compact cluster below the oesophagus (de Velasco et al., 2006), both proteins accumulate at tricellular contacts (Fig. 1A,A′, arrows) where Pyd colocalises with the tricellular junction markers Sidekick (Sdk; Letizia et al., 2019; Fig. 1B-B″) and non-muscle myosin II (see below). The distribution of Duf and Pyd changes during stage 14, when these proteins mobilise towards the equatorial cell cortex, which separates the two nuclei of the now binucleated nephrocytes, forming a ring (Fig. 1C-C‴, arrows). At stage 15, this sharp ring becomes distorted, presenting protrusions extending from it, when additional slit diaphragm complexes accumulate at locations close to their pre-existing positions (Fig. 1D-D″, arrows). Finally, at stage 16, slit diaphragm proteins are distributed in a filigree pattern throughout the external membrane of the nephrocyte exposed to haemolymph, reflecting the formation of slit diaphragms (as shown below by TEM analyses), although they are still less densely packed than in larval nephrocytes (Fig. 1E-E″, arrows; Carrasco-Rando et al., 2019).

The transition of slit diaphragm proteins from tricellular contacts to the equatorial cortex coincides with nephrocyte binucleation, suggesting a functional link between these two processes. We investigated whether binucleation results from a process of incomplete cytokinesis or from cell fusion. During *Drosophila* embryogenesis, the first 13 mitoses are synchronous and, after cellularisation occurs, most cells undergo three additional divisions within the next 4 h (until early stage 11; Edgar and O'Farrell, 1990). Close examination of 4′,6-diamidino-2-phenylindole (DAPI) staining (Fig. 1F-F‴) and staining for the mitotic marker phosphorylated histone H3 (PH3) (Fig. 1G-G‴) in *sns-GCN-nGFP* embryos expressing GFP in the nuclei of nephrocytes (Zhuang et al., 2009) reveal the presence of mitotic figures in nephrocytes at stage 13 (arrowheads in Fig. 1F-G‴), indicating that they have initiated an extra mitosis after the three post-blastoderm cell divisions. Furthermore, in *sns-GCN-nGFP* dividing nephrocytes, GFP accumulates throughout the cytoplasm, probably due to the breakdown of the nuclear envelope (arrows in Fig. 1F‴,G″), in contrast to its nuclear confinement in binucleated nephrocytes at later developmental stages (arrows in Fig. 1H,I). These observations are consistent with the interpretation that nephrocyte binucleation results from acytokinetic cell divisions, rather than from a process of cell fusion. Accordingly, one would expect garland nephrocytes to be binucleated in cell fusion mutants, whereas they would be mononucleated if cell division is blocked. Indeed, embryos deficient for the slit diaphragm proteins Duf [*Df(1)w^67k30^*, also removing *duf* paralogue *roughest*] and Sns (*sns^XB3^*), both presenting a complete block of myoblast fusion (Bour et al., 2000; Ruiz-Gómez et al., 2000; Fig. S1A,B,C, compare with Fig. S1D,E), exhibit binucleated garland nephrocytes (Fig. S1B′,C′; see also Weavers et al., 2009). In contrast, in zygotic *CycA^C551^* mutant embryos, which do not undergo mitoses beyond cell cycle 15 (Lehner and O'Farrell, 1989) and display syncytial muscles (Fig. S1F,G, compare with Fig. S1D,E), garland nephrocytes are mononucleated (Fig. S1G′). In sum, we conclude that binucleation of garland nephrocytes is due to a process of incomplete cytokinesis and suggest that the site of slit diaphragm proteins accumulation, at the equator of stage 14 nephrocytes, might be marked by prior positioning of a cytokinetic ring.

### Accumulation of slit diaphragm proteins at the equatorial cortex triggers slit diaphragm formation in garland nephrocytes

We then resorted to ultrastructural analyses to further elucidate how the changes in slit diaphragm protein distribution observed by confocal microscopy correlate with the actual assembly of slit diaphragms. We focused on examining transmission electron microscopy (TEM) sections taken at a plane containing both nuclei in binucleated nephrocytes. Representative examples of such images are shown for stage 14 (Fig. 1J), stage 15 (Fig. 1K) and stage 16 (Fig. 1L) embryos. In 80% of the stage 14 nephrocytes analysed we observed deep ingressions of the plasma membrane penetrating the nephrocyte at the level between the nuclei (arrowheads in Fig. 1J′). Continuity of the cytoplasm between sibling nuclei was observed in the most medial sections (asterisk), and in all cases membrane invaginations were sealed on the outside by slit diaphragm structures (arrows in Fig. 1J′ and inset). Similarly, 74% of the binucleated stage 15 nephrocytes presented deep membrane invaginations separating both nuclei (arrowheads in Fig. 1K′), but at this stage the lumina of the invaginations were wider than at stage 14, as if they were filled with haemolymph, and the winding membrane invaginations were sealed by multiple slit diaphragms (arrows). Similar void spaces between sibling nuclei were evident in *sns-GCN-nGFP* stage 15 nephrocytes (arrowheads in Fig. 1I), ruling out the possibility that they represent artefacts due to sample preparation. Stage 16 nephrocytes (Fig. 1L,L′) exhibited a partial regression of the membrane grooves, which appeared to be shorter and thinner, and, in 72% of them, it was common to observe scattered membranes running between sibling nuclei, which may represent the scars of previous grooves (asterisks). Additional membrane ingressions form channels close to the cell periphery. In all cases slit diaphragms seal the entrance to the channels (arrows).

**Fig. 2.**
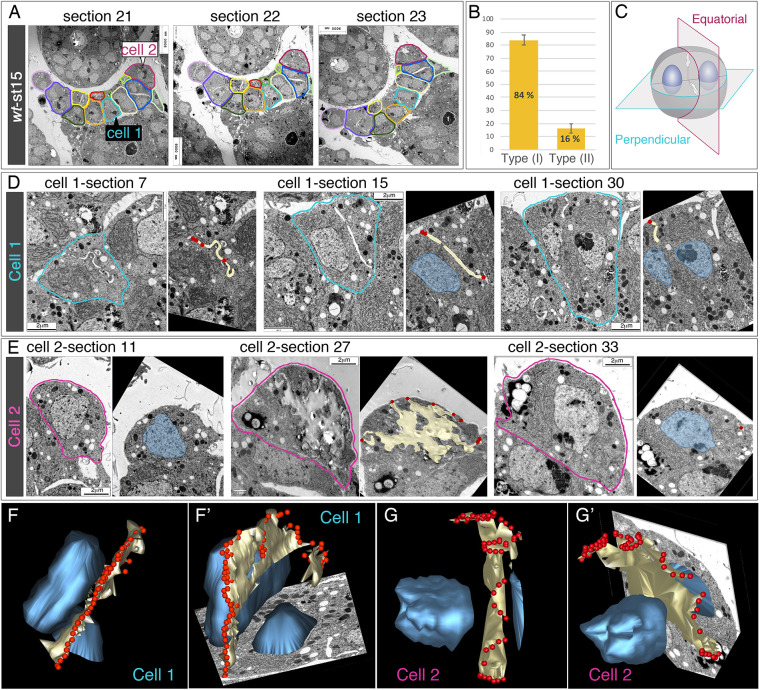
**3D reconstructions of the distribution of slit diaphragms at the equatorial cortex of binucleated embryonic garland nephrocytes.** (A) Selected progressive TEM micrographs from a string of garland nephrocytes at stage 15. Each nephrocyte is outlined in a different colour through the whole series. (B) Quantification of the two types of garland nephrocytes (I and II) found in wild-type embryos (see also Fig. S2). Data are mean±s.e.m. (C) Diagram of cell-sectioning perpendicular to the equatorial plane, cutting the nephrocyte between the two nuclei (these sections include both nuclei, cell 1) or through the equatorial plane (cell 2). (D,E) Selected progressive TEM images and the corresponding segmentations for cells 1 (D) and 2 (E); in the segmentations, nuclei are indicated in blue, lacunae formed by invaginated membranes in yellow and slit diaphragms in red. (F-G′) 3D reconstructions shown from two different viewpoints obtained for cells 1 (F,F′) and 2 (G,G′), showing the lacunae (yellow), the nuclei (blue) and the distribution of the first slit diaphragms assembled (red), which hold together both ends of the invaginated membrane and prefigure a ring that surrounds the cell in its equatorial plane. See also Movies 1-4.

To gain further insight into the global three-dimensional (3D) distribution of slit diaphragms, we focused on stage 15 embryos and analysed 33 serial TEM sections across a cluster of 13 nephrocytes. We followed each cell using a colour code (Fig. 2A). Visual inspection of serial sections pointed out the existence of two cell types (see also Fig. S2): type I, similar to the cell depicted in Fig. 1K, in which we observed the presence of membrane invaginations between the sister nuclei, which we call equatorial wedges, and a second type (II) in which the nuclei appeared to be closely apposed and membrane ingressions were mostly found on the outer membrane facing haemolymph. As type I nephrocytes were the most abundant (quantification in Fig. 2B), next we focussed on two cells of this group, cut at perpendicular planes (Fig. 2C). We performed manual alignment and segmentation (Fig. 2D,E) of the sections to generate 3D reconstructions, which are described below (Fig. 2F-G′; Movies 1-4). The outermost sections of cell 1 (cut perpendicular to the equatorial plane) showed a groove, sealed at its outer margins with diaphragms (Fig. 2D, section 7). In subsequent sections containing the nuclei, they were always located one on each side of the groove (Fig. 2D, sections 15, 30). On the other hand, in cell 2 (cut equatorially) the outermost sections lacked slit diaphragms, whereas consecutive sections included sequentially one nucleus, an irregular region with very low electron-density, centrally located, that exhibited slit diaphragms where it reached the outer cell membrane, and the second nucleus (Fig. 2E, sections 11, 27, 33). Analyses of the 3D reconstructions revealed that the furrows created by the ingression of the cell membrane always occurred along the equatorial cell plane that separates both nuclei, creating a hollow space (lacuna) in the middle, but keeping cytoplasmic connections between both cell halves. Furthermore, they confirmed that the ring-shaped accumulations of slit diaphragm proteins at the equatorial cortex, revealed by immunostainings, correspond to the assembly of the first slit diaphragms that are formed during development, which join on its outer side both edges of the deep membrane infoldings formed at this position (Fig. 2F-G′).

### An acytokinetic cell division creates membrane asymmetries in nephrocytes that determine the sites for slit diaphragm assembly

Vertebrate slit diaphragm proteins are found in lipid raft microdomains enriched in phosphatidylinositol(4,5)biphosphate (PIP2) (Arif et al., 2014). As we have shown the translocation of slit diaphragm proteins from tricellular contacts to the equator of dividing nephrocytes, and PIP2 accumulates at the cleavage furrow in dividing cells (Emoto et al., 2005; Field et al., 2005), it was tempting to speculate that the acquisition of the extra mitosis in garland nephrocytes would break the initial membrane symmetry typical of mesenchymal cells by creating a local enrichment in PIP2 at the site of assembly of a cytokinetic ring that would set up a favourable environment for the recruitment of slit diaphragm proteins and the subsequent assembly of slit diaphragms.

**Fig. 3.**
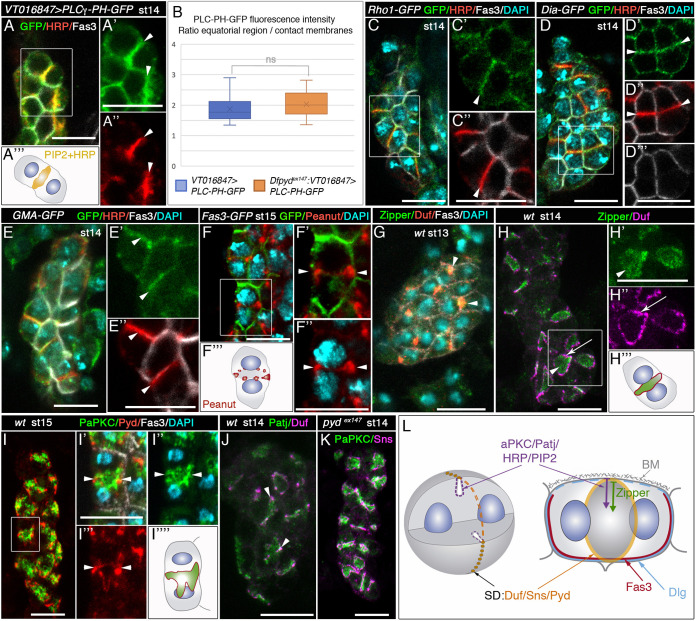
**Acytokinetic cell divisions break membrane symmetry in embryonic garland nephrocytes by generating PIP2-enriched microdomains.** (A-K) Single sections (A,C-E) and confocal *z*-projections (F-K) of embryonic garland nephrocytes of the specified developmental stages and genotypes, stained with the indicated antibodies. (A-A‴) In nephrocytes expressing *PLCγ-PH-GFP*, PIP2 accumulates at the equatorial plane, colocalising with the HRP epitope [arrowheads in magnified views A′,A″, *n*=12 nephrocytes (N)/2 strings (S), schematic in A‴]. (B) Quantification of *PLCγ-PH-GFP* fluorescence intensity per area in wild-type (Fig. 3A′) and *pyd^ex147^* (Fig. 4C′) backgrounds (N=24/S=4 and N=21/S=6, respectively). Data show the ratio between the signal at the equatorial region and that at the site of contact between adjacent nephrocytes. Box plot shows median values (horizontal bars), the mean values (indicated by X) and the first to third interquartile ranges (boxes); whiskers extend to the minimum and maximum data values. ns, not significant. (C-E″) GFP-tagged versions of Rho1 (C,C′, *n*=25N/6S), Dia (D,D′, *n*=44N/3S) and GMA (E,E′, *n*=24N/7S) accumulate at the equatorial region of stage 14 nephrocytes, colocalising with the HRP epitope (arrowheads in C′,C″,D′,D″,E′,E″). Note, this region is devoid of Fas3 (C″,D‴,E″). (F-F‴) Peanut localises at the nephrocyte equatorial cortex (arrowheads in magnified views F′,F″, schematic in F‴, *n*=32N/4S). (G-H‴) Zipper mobilises from tricellular contacts, where it colocalises with Duf, in mononucleated nephrocytes (G, arrowheads, *n*=30N/2S) to the equatorial plane of binucleated nephrocytes (H,H′, arrowheads, *n*=77N/5S; see Fig. S4). Simultaneously, Duf accumulates at the equatorial cortex (H,H″, arrows, schematic in H‴; see also Fig. 2F-G′, Fig. S4). (I-J) PaPKC (I-I″, *n*=160N/11S) and Patj (J, *n*=45N/4S) accumulate at the equatorial region of binucleated nephrocytes, partially colocalising with Pyd (I) and Duf (J) at the cortex (arrowheads, I′-I‴,J). (K) PaPKC and Sns distribution do not change in the absence of slit diaphragms (*pyd^ex147^* mutants, *n*=32N/3S). (L) Diagram showing the subcellular distribution in binucleated stage 14 nephrocytes of the indicated proteins and PIP2. Slit diaphragm components (in orange) accumulate in a ring at the equatorial cortex, Zipper (green arrow) only in the septum and apical determinants, the HRP epitope and PIP2 at the septum and at the equatorial cortex (purple arrow), colocalising in the latter region with slit diaphragm components. Scale bars: 10 µm.

**Fig. 4.**
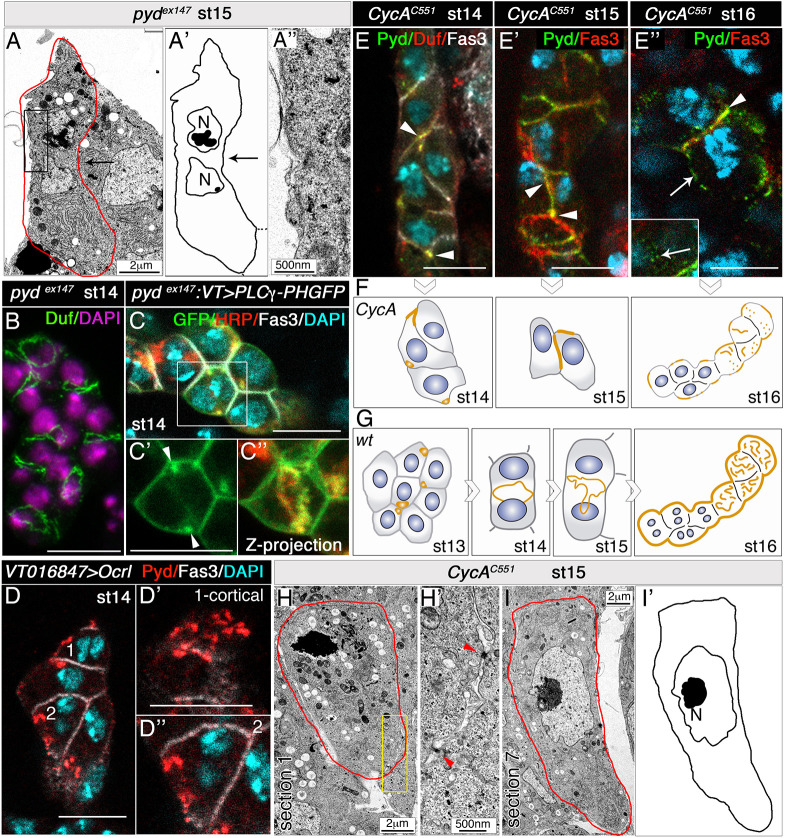
**Establishment of PIP2-enriched membrane domains accelerates slit diaphragm assembly.** (A-A″) TEM image, schematic and detail of stage 15 *pyd^ex147^* nephrocytes [*n*=12 nephrocytes (N)/3 embryos]. Note the absence of equatorial membrane invaginations (black arrows) and slit diaphragms (compare with Figs 1K,K′ and 2D-G). (B) Confocal *z*-projection of *pyd^ex147^* nephrocytes, showing Duf accumulation at the equatorial cortex [*n*=81N/5 strings (S)]. (C-C″) In *pyd^ex147^* nephrocytes expressing *UAS-PLCγ-PH-GFP* (*n*=21N/6S), devoid of membrane invaginations, PIP2 accumulates in a ring in the equatorial cortex (C″, arrowheads in C′, quantification in Fig. 3B; C,C′ single sections). (D-D″) Overexpression of *Ocrl* induces the premature localisation of Pyd at the nephrocyte outer membrane (D′, cell 1, cortical view; D″, cell 2, medial view, *n*=29N/4S; compare with Fig. 1C-C‴,E-E″). (E-I′) Individual optical sections (E,E″), *z*-projection (E′), schematics (F,I′) and TEM images (H-I) of *CycA^C551^* nephrocytes at the indicated embryonic stages, and schematic of control nephrocytes (G). When the extra acytokinetic cell division is prevented, Duf and Pyd accumulate at tricellular contacts between mononucleated nephrocytes at stages 14 (*n*=18N/3S) and 15 (*n*=18N/4S) (arrowheads in E,E′, compare with Figs 1A,C,D and 4G). At stage 16, Pyd still localises to cell contacts in some nephrocytes (E″, arrowhead; *n*=6N/3S), and scattered Pyd distribution can be observed at nephrocyte membranes exposed to haemolymph (arrows in E″, the inset shows a superficial view; compare with Fig. 1E). Nuclei (DAPI staining) are shown in blue in E-E″. Unlike the wild-type, stage 15 *CycA* nephrocytes lack slit diaphragms (H-I′), although occasionally electron-dense material is observed between adjacent nephrocytes (H′, red arrowheads). Scale bars: 2 µm (A,H,I); 500 nm (A″,H′); 10 µm (B-E″).

To test this hypothesis, we first monitored the accumulation of PIP2 in nephrocytes. We used the UAS-Gal4 system to express the PIP2 sensor PLCγ-PH-GFP (Pinal et al., 2006) (using *VT016847-Gal4*; Fig. S3B-B″) from embryonic stage 12 in nephrocytes, finding it to exhibit a robust accumulation at the equatorial wedge in stage 14 nephrocytes, an area that is also enriched in glycoproteins carrying the carbohydrate epitope recognised by anti-HRP (Fig. 3A-A‴, arrowheads; Figs S2 and S3A). To rule out that the high intensity of the GFP signal at the equatorial wedge was due to the presence of two apposed membrane surfaces, we compared signal intensity at the equatorial wedge and at the site of cell-cell contacts in wild-type nephrocytes, both sites containing two closely apposed membranes. As shown in Fig. 3B, signal intensity at the equator was double that at cell contacts. Similar results were obtained in *pyd^ex147^* mutant nephrocytes, where equatorial membrane ingressions did not form (Figs 3B and 4A,C-C‴; see below). As PIP2 regulates the activity and/or promotes the recruitment to the equatorial region of various proteins required for cytokinesis (Logan and Mandato, 2006; Rousso et al., 2013; Yoshida et al., 2009), we examined whether proteins associated with the assembly of a cytokinetic actomyosin ring also accumulate in this region. Indeed, we found that the following proteins accumulated at the nephrocyte equatorial cortex: (1) Rho1, the Rho GTPase that regulates contractile ring assembly; (2) the formin Diaphanous (Dia), the downstream effector of Rho1 that nucleates filamentous actin; (3) F-actin, which is recognised by the accumulation of GFP fused to the actin-binding domain of Moesin (*sGMA-GFP*) or by *UAS-Lifeact-GFP*; and (4) the *Drosophila* septin Peanut, which is involved in establishing a link between the contractile ring and the plasma membrane, and the localisation of which at the cytokinetic cleavage furrow depends on PIP2 and Anillin (Cabernard, 2012; D'Avino et al., 2008; Goldbach et al., 2010) (Fig. 3C-F‴; Fig. S3C-D′). Furthermore, we also observed that Zipper, non-muscle myosin II heavy chain, which must be localised to the site of future cleavage before the assembly of the contractile ring, mobilised from tricellular contacts, where it accumulates along with Duf at stage 13 (Fig. 3G) towards the equatorial cell plane at stage 14 (Fig. 3H-H‴; Fig. S4). Thus myosin II/Zipper follows the same path as the slit diaphragm proteins, although whereas these proteins are located in the outer membrane, myosin II is found throughout the equatorial wedge (Fig. 3H-H‴; Fig. S4). Of note, the distribution of F-actin and myosin II does not change from stages 14 to 15 (Fig. 3E-E″,H-H″; Fig. S3E-F′) as would be expected if the constriction of the contractile ring had occurred.

In addition, we reasoned that, being an apical identity determinant, PIP2 would also enable the compartmentalisation of apical membrane proteins such as aPKC and Patj (a member of the apical complex Crumbs/Stardust/Patj; Buckley and St Johnston, 2022). Consistently, both proteins were also delivered to the nephrocyte equatorial region (Fig. 3I-J). Conversely, the basolateral protein Discs large (Dlg, Buckley and St Johnston, 2022) is excluded from this membrane domain, and is distributed throughout the rest of the nephrocyte membrane (Fig. 3L; Fig. S3G,G′), similar to Fas3.

Furthermore, we reasoned that if the accumulation of slit diaphragm proteins at the nephrocyte equatorial cortex depends on their stabilisation at PIP2-enriched microdomains, then they will be maintained in these membrane domains even when the assembly of slit diaphragms is compromised, as in *pyd* mutants (Carrasco-Rando et al., 2019). Consistently, in *pyd*-deficient embryos, which neither form slit diaphragms (Fig. 4A-A″) nor present membrane ingressions between sibling nuclei (Fig. 4A,A′, compare with Fig. 1K,K′), Duf still accumulates at the equatorial cell cortex at stage 14 (Fig. 4B), which is highly enriched in PIP2 (Fig. 4C-C″, quantified in Fig. 3B).

In addition, experimental modification of PIP2 levels in nephrocytes would be expected to have an impact on slit diaphragm assembly. Therefore, we decided to modify PIP2 levels by manipulating the Inositol 5-phosphatase Ocrl, which dephosphorylates PIP2 (Ben El Kadhi et al., 2011; Del Signore et al., 2017). Depletion of Ocrl in *Drosophila* S2 cells leads to enrichment of PIP2 on endomembranes, inducing the ectopic accumulation of cytokinetic effectors at these membranes (Ben El Kadhi et al., 2011). Analysis of the effect of Ocrl loss in nephrocytes was hampered by the ability of null *Ocrl^Δ3^* mutants to complete embryogenesis, surviving until larval or pupal stages probably due to persistent maternal activity. The use of RNAi-mediated attenuation was not possible, as it does not work efficiently during embryogenesis. In addition, optogenetic manipulation of PIP2 levels is precluded in embryonic nephrocytes by their location, floating freely in haemolymph. Therefore, we resorted to increase PIP2 levels in nephrocytes by overexpressing *UAS*-*Ocrl* with *VT016847-Gal4*. As shown in Fig. 4D, these nephrocytes showed premature accumulation of the slit diaphragm components Duf and Pyd in patches on the outer nephrocyte membrane in stage 14 embryos, when in wild-type embryos they were confined to the equatorial cortex (Fig. 1C-C‴).

Overall, these results strengthened our initial hypothesis that the triggering of slit diaphragm formation at specific membrane locations in mesodermal nephrocytes, which otherwise would not be polarised, is due to the creation of a favourable membrane environment by an acytokinetic cell division. The PIP2-enriched domains generated would support the stabilisation of the slit diaphragm main components. Accordingly, prevention of the acytokinetic cell division should affect the formation of slit diaphragms.

### Failure to complete cytokinesis prolongs local membrane asymmetries accelerating the assembly of slit diaphragms

**Fig. 5.**
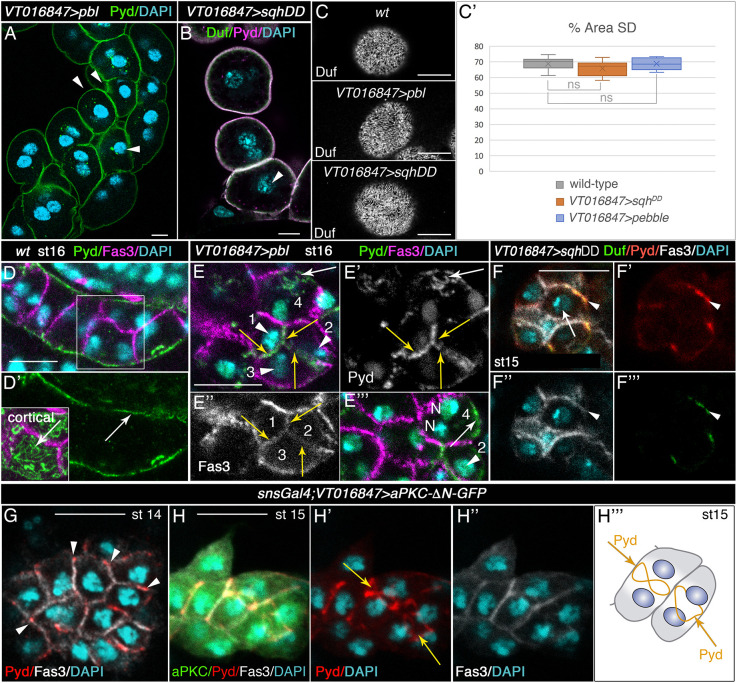
**Promoting ring constriction in the nephrocyte extra acytokinetic mitosis delays the assembly of slit diaphragms.** (A-C′) Individual confocal sections (A,C), *z*-projection (B) and quantification (C′) of larval nephrocytes of the indicated genotypes stained with antibodies against slit diaphragm proteins. Arrowheads point to mononucleated nephrocytes (*n*=88 nephrocytes (N)/12 strings (S) out of 294N in A and *n*=8N/5S out of 67N in B], which at this stage exhibit a normal density of slit diaphragms [C, Duf staining, external views, quantified in C′ as percentage of nephrocyte surface area covered by slit diaphragms (SD); wt, *n*=16 regions of interest (ROIs), 9 cells, 5 strings; *VT016847>sqh^DD^*, *n*=11 ROIs, 5 cells, 3 strings; *VT016847>pbl*, *n*=12 ROIs, 8 cells, 5 strings; mean±s.d.]. Box plot shows median values (horizontal bars), mean values (indicated by X) and first to third interquartile ranges (boxes); whiskers extend to the minimum and maximum data values. ns, not significant. (D-F‴) *Z*-projections (D-E″) and single sections (E‴-F‴) of embryonic nephrocytes of the indicated genotypes. Note in *pbl*-overexpressing nephrocytes the colocalisation of Pyd and Fas3 (yellow arrows in E-E″) at the cell membrane between adjacent mononucleated nephrocytes 1, 2 and 3 (arrowheads in E,E‴) and Pyd localisation at the outer membrane (white arrow in E,E′ and in a different plane in E‴) in binucleated cell 4 (*n*=13N/6S). In F-F‴ (*n*=7N/5S), arrowheads point to the colocalisation of Duf and Pyd with Fas3 at contact membranes abutting mononucleated nephrocytes overexpressing *sqh^DD^* (arrow in F). (G-H‴) Ubiquitous overexpression of a constitutively active form of aPKC in embryonic nephrocytes delays the onset of the acytokinetic cell division to stage 15. At stage 14 nephrocytes are mononucleated (G, *n*=40N/5S) and accumulate slit diaphragm proteins at tricellular contacts (arrowheads). At stage 15 (H-H‴, *n*=20N/7S), slit diaphragm proteins accumulate at the equatorial cortex in binucleated cells (arrows). N, nucleus (E‴). Scale bars: 10 µm.

We propose that the process of incomplete cytokinesis would help to create PIP2-enriched membrane domains and extend the period of time during which such asymmetry is maintained in garland nephrocytes. To confirm this hypothesis, we sought to experimentally challenge the system, anticipating the expected outcome to different scenarios. First, prevention of the acytokinetic cell division should affect slit diaphragm formation. Indeed, in *CycA* mutant embryos, which have mononucleated nephrocytes (Fig. S1G′), the mobilisation of slit diaphragm proteins from tricellular contacts to the equatorial cell cortex was delayed, as they were still concentrated in cell contacts at stages 14 and 15 (Fig. 4E-F, compare with Figs 1C-E and 4G) and, congruently, slit diaphragms were hardly present at the end of embryogenesis (Fig. 4H-I′). Second, we anticipated that completing cytokinesis of the extra post-blastoderm mitosis should delay slit diaphragm assembly in garland nephrocytes. During cytokinesis, the RhoGEF Pebble (Pbl, the fly orthologue of ECT2) activates Rho1 at the contractile ring that, in turn, activates Drok (also known as Rok), which directly phosphorylates the regulatory light chain of non-muscle myosin II, Spaghetti squash (Sqh), at serine 20 and threonine 21, promoting contractile ring assembly and contraction (reviewed by Cabernard, 2012). As our data suggested a possible failure in the constriction of the actomyosin ring during the nephrocyte acytokinetic mitosis (Figs S3H-H‴, S3E-F′), we attempted to promote ring constriction in nephrocytes by overexpressing either Pbl or the constitutively active form of Sqh (*UAS-sqh^DD^*, a phosphomimetic form of Sqh; Mitonaka et al., 2007). In both examples, garland nephrocytes were able to complete cytokinesis of the extra mitosis, although with low penetrance, as shown in third instar larvae by the presence of nephrocytes containing a single nucleus that are usually smaller than binucleated nephrocytes (arrowheads in Fig. 5A,B). These mononucleated nephrocytes exhibited a normal distribution of slit diaphragm proteins in third instar larvae (Fig. 5C, quantified in C′), but the assembly of slit diaphragms was delayed during embryogenesis, as shown by the distribution of Pyd at embryonic stage 16. Thus, although in the wild-type Pyd and other slit diaphragm components were distributed in a filigree pattern at the membrane exposed to the haemolymph (Fig. 5D,D′ and inset, arrows) and excluded from the Fas3-labelled cell contact membranes (Figs 1E,E′ arrowheads, 5D,D′), in experimental nephrocytes overexpressing *pbl* we observed membranes located between adjacent nephrocytes accumulating Fas3 and high levels of Pyd (yellow arrows in Fig. 5E-E″), and in all cases these membranes were contributed by mononucleated nephrocytes (arrowheads in Fig. 5E,E‴). Note, however, that due to the low penetrance of ring constriction induction mentioned above, a large number of experimental nephrocytes were binucleated (cell 4 in Fig. 5E,E‴) and exhibited the characteristic distribution of Pyd at the haemolymph-exposed membrane (arrow in Fig. 5E,E′,E‴). Similar results were obtained for *sqh^DD^* overexpression (Fig. 5F-F‴). These results strongly suggest that, as in *CycA* mutants, slit diaphragm formation in these mononucleated nephrocytes is delayed during embryogenesis.

Altogether, the results presented in this work lead us to conclude that the mechanism that accelerates slit diaphragm assembly in garland nephrocytes derives from the acquisition of an additional post-blastoderm acytokinetic cell division, which helps to polarise these mesodermal cells by generating and maintaining PIP2-enriched membrane heterogeneities that favour the concentration of slit diaphragm proteins, thus sustaining the assembly of slit diaphragms. Consequently, garland cells are the only functional nephrocytes in embryos and early larvae, as revealed by their unique ability to uptake dextrans from the circulating haemolymph (Fig. S3H,I).

Another relevant question is the role that apical-basal polarity proteins play in slit diaphragm formation. There is compelling evidence that these proteins play a fundamental role in compartmentalising the epithelial cell membrane by creating membrane microdomains that organise the distribution of different cellular effectors (Martin et al., 2021). We investigated whether polarity proteins could also be instrumental in nephrocytes for determining the site of silt diaphragm assembly. We have observed that the apical determinants aPKC (revealed by staining with anti p-Thr555-aPKCz antibody, which recognises activated aPKC; Wirtz-Peitz et al., 2008) and Patj accumulate at the equatorial plane partially overlapping with slit diaphragm markers (Fig. 3I-J). We anticipated two possible scenarios compatible with our observations. One was that the establishment of PIP2-enriched domains in the equatorial cortex of garland nephrocytes drives the accumulation of slit diaphragm proteins and apical polarity determinants independently of each other, and thus apical proteins do not provide information for the positioning of slit diaphragms. Conversely, the apical proteins, could participate directly in targeting slit diaphragm proteins to their final destination by modifying the composition of the membrane. In this latter case, we should expect changes in the distribution of slit diaphragm proteins by manipulating the activity of aPKC. In embryonic garland nephrocytes, we overexpressed a constitutively active form of aPKC (aPKC^ΔN^-GFP, Tian and Deng, 2008) and a dominant-negative kinase-dead variant of aPKC that was targeted to the cell membrane by addition of a CAAX moiety (DaPKC^CAAXDN^; Sotillos et al., 2004), which were previously used in epithelial cells and in larval nephrocytes (Heiden et al., 2021; Sotillos et al., 2004; Tian and Deng, 2008; Fig. S5A-D). Overexpression of DaPKC^CAAXDN^ did not overtly modify the localisation of slit diaphragm proteins, nor the timing of the binucleation process (Fig. S5E-E″). Interestingly, the overexpression aPKC^ΔN^, homogeneously distributed in the membrane, nuclei and cytoplasm of nephrocytes (Fig. 5H; see also Tian and Deng, 2008) induced a delay in their acytokinetic division, as they remain mononucleated until stage 15 (Fig. 5G-H‴). We do not know how, mechanistically, overexpression of aPKC^ΔN^ impinges on the timing of cell division, though it may be related to its nuclear localisation, as it has been reported that nuclear aPKC regulates cell cycle progression (Drummond and Prehoda, 2016). However, despite such delay, once cells became binucleated, we did not observe the expected redistribution of Duf and Pyd to the whole cell contour indicative of an instrumental role of aPKC in targeting slit diaphragm proteins to specific membrane regions. Instead, they remained localised in the equatorial cortex (Fig. 5H-H‴). These results, together with the observation that aPKC accumulates at the equatorial region in stage 14 *pyd^ex147^* nephrocytes, lacking slit diaphragms (Fig. 3K), are consistent with the hypothesis that slit diaphragm proteins and apical polarity determinants accumulate in PIP2-enriched domains independently of each other.

## DISCUSSION

Slit diaphragms are modified cell junctions, conserved from flies to humans, that work as molecular filters during the processes of haemolymph/blood ultrafiltration. It has been extensively documented, both in vertebrates and flies, that junctional remodelling between occludens/septate-like junctions and slit diaphragms is recurrently associated with the processes of slit diaphragm development, injury and repair (Carrasco-Rando et al., 2019; Ichimura et al., 2016; Kriz et al., 2013), and that the slit diaphragm component ZO1/Pyd and the apical determinant aPKCλ colocalise in the junctional complex that precedes the formation of the first slit diaphragms in podocytes (Huber et al., 2009; Schnabel et al., 1990). However, in spite of these observations, the precise mechanisms that direct the assembly of slit diaphragms have not yet been fully elucidated.

In this study we use the embryonic garland nephrocytes to address this relevant question. Interestingly, garland nephrocytes differ from pericardial nephrocytes by being binucleated and the only ones that assemble slit diaphragms during embryogenesis. As, contrary to podocytes, nephrocytes are mesodermal in origin and do not show any sign of apical-basal polarity, it was challenging to determine how symmetry is broken in these cells to define discrete membrane microdomains that allow the stabilisation and accumulation of slit diaphragm proteins required to trigger diaphragm assembly.

Our data suggest that the event that generates membrane asymmetry in garland nephrocytes is the acquisition of an extra post-blastoderm mitosis lacking cytokinesis, which results in the binucleation of garland nephrocytes. We show that coinciding with this acytokinetic mitosis, at embryonic stage 14, slit diaphragm components, including Duf and Pyd, mobilise from tricellular contacts, where they colocalise with myosin II and Sidekick at stage 13, to the nephrocyte equatorial membrane located between the two nuclei of the nephrocyte. This equatorial membrane has characteristics typical of a cleavage furrow membrane, which differentiate it from the rest of the nephrocyte membrane. Thus, we found it to accumulate PIP2, Rho1, Dia, F-actin, myosin II and Peanut, all suggestive of the assembly of an actomyosin cytokinetic ring, while it is deprived of Fas3. PIP2-enriched domains not only play key roles at the plasma membrane of the cytokinetic furrow by regulating cytoskeleton dynamics and maintaining the association of several cytokinetic proteins (Echard, 2012), but also facilitate the partition of several membrane-associated proteins, including apical determinants and slit diaphragm components (Gass et al., 2022; Wills and Hammond, 2022). Indeed, both aPCK and Pyd are targeted to PIP2-enriched membrane domains through their polybasic and PDZ domains, respectively (Dong et al., 2020; Ivarsson et al., 2011). As both proteins migrate together from the apical to the basal portion of podocytes before the transition from occludens junctions to slit diaphragms during development (Ichimura et al., 2016; Reeves et al., 1978), we investigated whether aPKC plays an instructive role providing positional information for slit diaphragm assembly in nephrocytes. Our data suggest that this is not the case, as overexpression of constitutively-active aPKC^ΔN^, homogeneously distributed in the membrane, nuclei and cytoplasm of nephrocytes, does not alter the localisation of slit diaphragm proteins, which continue to accumulate in the equatorial cortex of the nephrocyte, thus pointing to PIP2 as the main determinant of slit diaphragm assembly sites. This interpretation does not, however, exclude that apical proteins may have an important role in regulating slit diaphragm stability and recycling as have been proposed in multiple studies (Heiden et al., 2021; Hochapfel et al., 2017; Huber et al., 2009; Koehler et al., 2022; Satoh et al., 2014).

Technical limitations have prevented us from studying the consequences of PIP2 depletion on the accumulation of slit diaphragm components at the equatorial cortex in embryonic nephrocytes. However, we have determined that the overexpression of Ocrl in these cells from stage 12, which should increase PIP2 levels, led to a premature accumulation of slit diaphragm proteins in the outer nephrocyte membrane, thus indicating that PIP2-enrichment directly affects the stabilisation of the diaphragm components in flies. Therefore, we hypothesise that the acquisition of the extra post-blastoderm acytokinetic mitosis in garland nephrocytes would generate and maintain membrane domains enriched in PIP2, facilitating the concentration and/or organisation of slit diaphragm proteins such as Sns and Duf and triggering slit diaphragm assembly. In our hypothesis, the prevention of cytokinesis becomes crucial, as it delays the resolution of the cytokinetic ring and thus prolongs the time during which the PIP2 membrane asymmetry is maintained. Our results showing that forcing completion of cytokinesis in the extra post-blastoderm mitosis in garland nephrocytes delays slit diaphragm formation are in complete agreement with this interpretation.

Our observations allow us to propose the following scenario for the assembly of the first slit diaphragms driven by the acytokinetic mitosis. (1) The accumulation of F-actin and myosin II filaments together with those of the actin crosslinking septin Peanut, which participates in the linking of the actomyosin ring with the cellular membrane, may lead to the assembly of actomyosin bundles at the equatorial plane. They could induce small ingressions of the membrane as a result of the sliding of actin filaments on myosin II, which will shorten the bundles even in the absence of myosin contraction. (2) The maintenance of PIP2-enriched membrane domains in the equatorial cell cortex would favour the accumulation of slit diaphragm proteins at this position, leading to the subsequent assembly of nascent slit diaphragms, which hold together both ends of the initial membrane ingressions that follow the process of cytokinetic ring assembly. (3) Sealing of the edges of the invaginated membranes by the slit diaphragms would lead to the growth of the channels thus generated and the formation of the equatorial wedges, despite the fact that constriction of the cytokinetic ring does not occur. This interpretation is supported by the observation that equatorial wedges did not form in the absence of slit diaphragms, although there is an accumulation of myosin, F-actin, PIP2 and slit diaphragm components in the equatorial cortex. In addition, we propose that the first slit diaphragms formed at the equatorial cortex would also help maintain membrane heterogeneity by acting as a nucleation centre for the recruitment of additional slit diaphragm complexes, by a hitherto unknown mechanism, until eventually the entire nephrocyte outer membrane is covered with slit diaphragms. This hypothesis is based on the observation of the evolution of slit diaphragm proteins distributions from stage 14 to 15 (this work) and further supported by the temporal and spatial emergence of slit diaphragms in the rescue experiments described in Carrasco-Rando et al., 2019, where the first slit diaphragms appeared close to cell-cell contacts, and the next, close to the previous ones.

Therefore, we have proposed a plausible mechanism to account for the formation of slit diaphragms in garland nephrocytes during embryonic stages, based on the execution of an extra acytokinetic post-blastoderm division. But what could be the biological advantage of such an event? In other words, what improvement would the formation of slit diaphragms during embryogenesis bring to the organism? We would like to speculate that the acquisition of functional excretory cells competent in ultrafiltration during embryogenesis would be equivalent to the formation of the first vertebrate kidney, the pronephros, and that similarly to its function in plasma filtration and osmoregulation during embryonic and young free-swimming larvae in fish and amphibians, garland nephrocytes would help maintain haemolymph homeostasis during embryogenesis and early larval stages in *Drosophila*, as they are the only functional nephrocytes at these stages.

## MATERIALS AND METHODS

### *Drosophila* strains

The following stocks were used: *Oregon R*, *pyd^EX147^* (Djiane et al., 2011), *sns-GCN-nGFP* (Zhuang et al., 2009), *hand-GFP* (Han et al., 2006), *Fas3^MI03674^* (GFP-tagged Fas3), *Df(3L)vin3*, *CycA^C8LR1^*, *UAS-Lifeact-GFP* [Bloomington *Drosophila* Stock Center (BDSC), #59809, #6627, #2609 and #35544], *CycA^C551^* (this study, see below), *sdk^CPTI000337^*, *Rho1-GFP* (Kyoto *Drosophila* Stock Center, #115107 and #110833), *VT016847-Gal4* [Vienna *Drosophila* Resource Center (VDRC), #203048], *UAS-PLCγ-PH-GFP* (Pinal et al., 2006), *UAS-Venus-Pbl* (a gift from Stephen L. Gregory, University of Adelaide, Australia), *UAS-sqh^DD^* (Mitonaka et al., 2007), *Dia-GFP* (Schmidt et al., 2021), *sGMCA-GFP* (Dutta et al., 2002), *UAS-OCRL-GFP* (Del Signore et al., 2017), *UAS-aPKC^ΔN^-GFP* (Tian and Deng, 2008) and *UAS-aPKC^CAAXDN^* (Sotillos et al., 2004).

### Characterisation of the CycA^C551^allele

The strong loss-of-function allele *CycA^C551^* used in this work was generated by EMS mutagenesis and verified by lack of complementation with both *Df(3L)vin3* and *CycA^C8LR1^*. To sequence the lesion present in this allele, oligonucleotides CycA-ex1_3-5′: 5′-GCACTGCTGGAGTTGCCAGTCC, CycA-ex1_3-3′: 5′-GCGATGAAAGCGAGTTTGTGAG, CycA-ex4_7-5′: 5′-GCATCTTCCAGAAGAAACATCGCC and CycA-ex4_7-5′: 5′- GCTGACTGCGACCGAACTGCAATTG were used to amplify PCR products covering the *CycA* coding region from genomic DNA extracted from *CycA^C551^* homozygous mutant embryos, which were selected for lack of GFP expression present in the balancer chromosome *TM3 twi-Gal4::2x UASeGFP*. Sequencing of three independent PCR reactions revealed the presence of a missense mutation (G281D in CycA-PC) in the first cyclin box fold domain of CycA, which affects a highly conserved amino acid (Brown et al., 1995), as responsible for the mutant phenotype of *CycA^C551^*.

### Antibodies

The following antibodies were used in this work: guinea-pig anti-Duf extracellular (1:100) and rabbit anti-Duf extracellular (1:400; Weavers et al., 2009), rabbit anti-Pyd (1:100) and rat anti-PydEx5 (1:200; Carrasco-Rando et al., 2019), mouse anti-Fas3 [1:10; Developmental Studies Hybridoma Bank (DSHB), AB-528238], mouse anti-Peanut (1:50; DSHB, AB-528429) and mouse anti-Discs large (1:50; DSHB, AB-528203), rabbit anti-phospho-Histone H3 (Ser10) (1:100; Upstate/Sigma-Aldrich, 06-570), rabbit Cy3-anti-HRP (1:100; Jackson ImmunoResearch, AB_2340262), rabbit anti-GFP (1:300; Thermo Fisher Scientific, A-6455), rabbit anti-GFP (1:300; Chromotek, PABG1), rabbit anti-Zip (1:50; Kiehart and Feghali, 1986), rabbit anti-p-PKC (Thr410) (1:10; Santa Cruz Biotechnology, sc-12894-R), rabbit anti-PKCξ (C-20) (1:20; Santa Cruz Biotechnology, sc-216), rabbit anti-Patj (1:50; gift from Hugo Bellen, Bhat et al., 1999), rat anti-Sns (1:100; gift from Elizabeth Chen, Bour et al., 2000), anti-cubn2 (1:200; Atienza-Manuel et al., 2021) and anti-Sdk (1:200; Astigarraga et al., 2018). The following secondary antibodies were used: Alexa Fluor 488, 555 and 647 (Thermo Fisher Scientific) and DyLight 549 and 488 goat anti-rabbit IgGs (Vector Laboratories, DI-1549 and DI-1088). All secondary antibodies were used at 1:500 dilution.

### Immunohistochemistry

Immunostainings were performed with minor modifications to the protocols described in Ruiz-Gómez and Ghysen (1993) and Atienza-Manuel et al. (2021). For stainings with anti-Duf and anti-Pyd antibodies the samples were hot fixed as described in Carrasco-Rando et al. (2019). Stained embryos were examined after removal of the central nervous system in mounting medium. Images were obtained using a Zeiss AxioImager2 vertical microscope and confocal microscopes LSM900 and LSM710 (Zeiss). As indicated in the figure legends, some confocal images correspond to *z*-projections from a series of confocal sections.

### *In vivo* dextran uptake assay

Dechorionated stage 16 *sns-nGFP* or *hand-GFP* embryos were covered with oil 10S (VWR) and injected with 0.5 mg/ml Dextran-Alexa Fluor 568; 10,000 MW (Invitrogen D22912) diluted in injection buffer (5 mM KCl and 10 mM sodium phosphate buffer, pH 7.2). Embryos were kept at 25°C for 1 h after injection and then visualised using a LSM710 (Zeiss) confocal microscope.

### Electron microscopy

Dechorionated embryos were fixed for 10 min on a rocking platform in a 1:1 mixture of heptane and 12.5% glutaraldehyde in 0.1 M sodium phosphate buffer, pH 7.2, rinsed and transferred to double-sided sticky tape for manual devitellinisation. The embryos were then fixed for 1 h in fixative solution (4% paraformaldehyde, 2% glutaraldehyde and 2% tannic acid in 0.1 M sodium phosphate buffer, pH 7.2), washed and post-fixed for 1 h at 4°C with aqueous 1% OsO_4_. Post-fixed embryos were washed with distilled water and negatively stained with 1% aqueous uranyl acetate for 1 h, followed by washes in distilled water and dehydration through 50, 70, 95 and 100% acetone series. The embryos were embedded in epoxy resin (Epon812/dodecenyl succinic anhydride/methyl nadic anhydride/benzyl dimethylamine from TAAB). Thin sections (70 nm) were cut using an UltracutE (Leica) ultramicrotome, stained with uranyl acetate and 2% lead citrate before visualisation in a Jem1010 (JEOL) instrument working at 80 kV and in a JEM1400 Flash (JEOL) microscope working at 100 kV. For the 3D reconstructions shown in Fig. 2 and Movies 1-4, 70 nm-thick serial sections were cut, keeping alternate sections for later examination, up to a total of 33.

### 3D reconstructions

Electron microscopy images were manually aligned using the TrakEM2 tool from Fiji/ImageJ software. Segmentation and 3D reconstruction was performed using the IMOD/3dmod programme.

### Image and statistical analysis

Fiji software was used for image processing and quantification. Statistical analysis was performed using GraphPad Prism 7.0 trial software. For *PLCγ-PH-GFP* quantification, data are presented as the ratio between membranes of the same nephrocyte in order to normalise putative experimental variations due to differences in sample processing. Statistical significance was determined using a two-tailed unpaired *t*-test for *PLCγ-PH-GFP* fluorescence intensity quantification (Fig. 3B) and an ordinary one-way ANOVA test for slit diaphragm quantification (Fig. 5C′). *P*-values<0.05 were considered statistically significant. Data distribution was assumed to be normal, but this was not formally tested.

## Supplementary Material

10.1242/develop.201708_sup1 Supplementary information
