# Supplementary material for: An acytokinetic cell division creates PIP2-enriched membrane asymmetries leading to slit diaphragm assembly in Drosophila nephrocytes
Source: Development. 2023 Sep 18;150(18):dev201708. doi: 10.1242/dev.201708 (PMC10546876; doi:10.1242/dev.201708)
Supplement: Supplementary information [file develop-150-201708-s1.pdf]

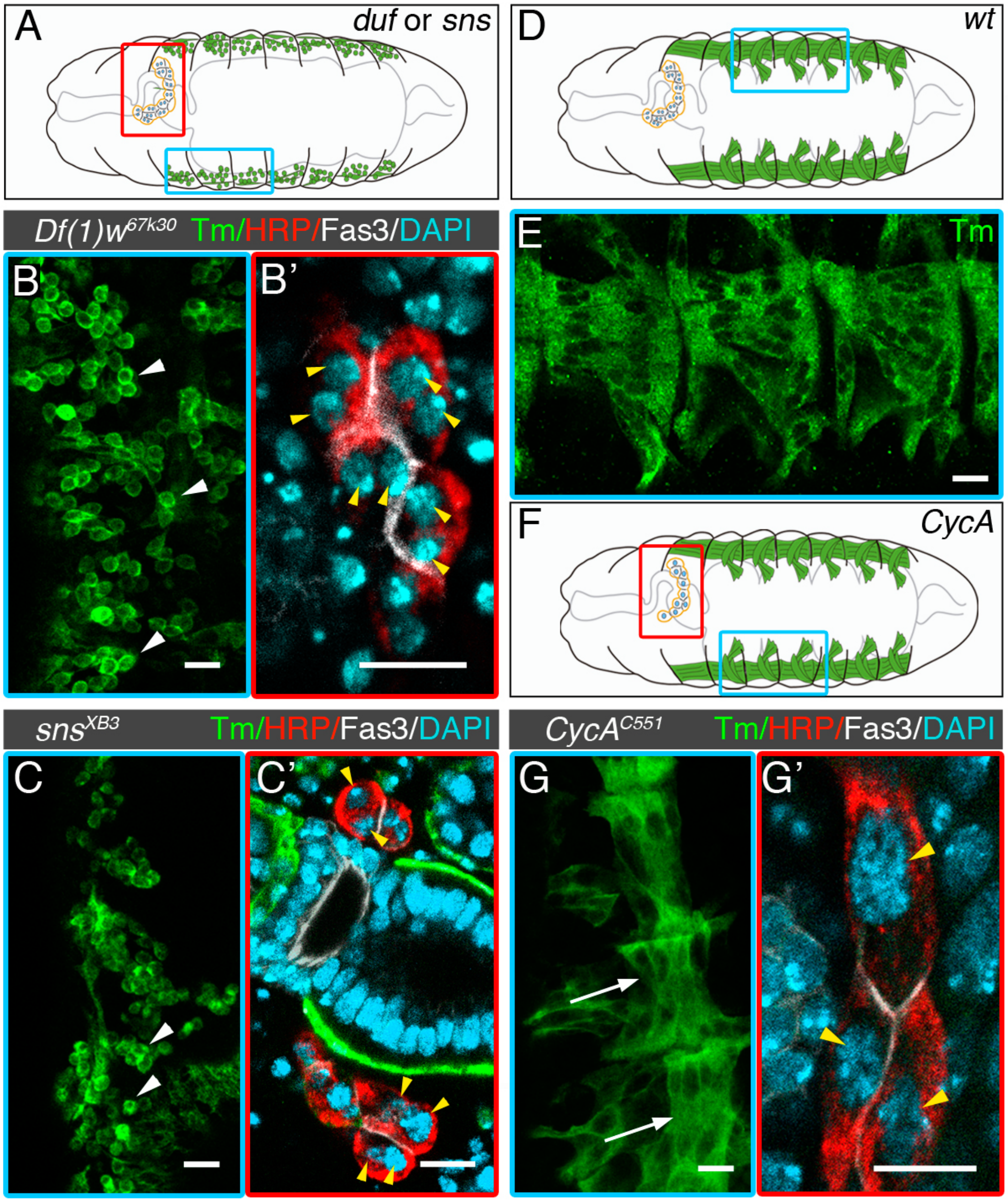

Fig. S1. Binucleation of garland nephrocyte is caused by an acytokinetic cell division. (A-G') Schematics (A, D, F) and confocal Z-projections (B-C, E, G) or single sections (C', G') of stage 16 embryos of the indicated genotypes stained with antibodies for Tropomyosin (Tm), HRP epitope and Fas3. The squares in the schemes show the regions magnified in the corresponding embryos. In embryos lacking *duf* and *rst* (*Df(1)w^{67k30}*, n = 46N/4S) or *sns* (*sns^{XB3}*, n = 156N/8S), there is a complete lack of myoblast fusion as revealed by the expression of Tm in unfused myoblasts (arrowheads in B, C), and the garland nephrocytes are binucleated (yellow arrowheads in B', C'), revealing that Duf and Sns are not required for nephrocyte binucleation. In contrast, in *CycA^{C551}* mutants (n = 91N/14S), myoblast fusion is unaffected, as evidenced by the presence of syncytial muscles (arrows in G), and they exhibit mononucleated garland nephrocytes (yellow arrowheads in G'), indicating that binucleation of nephrocytes correlates with cell divisions. Scale bars represent 10 µm.

Development • Supplementary information

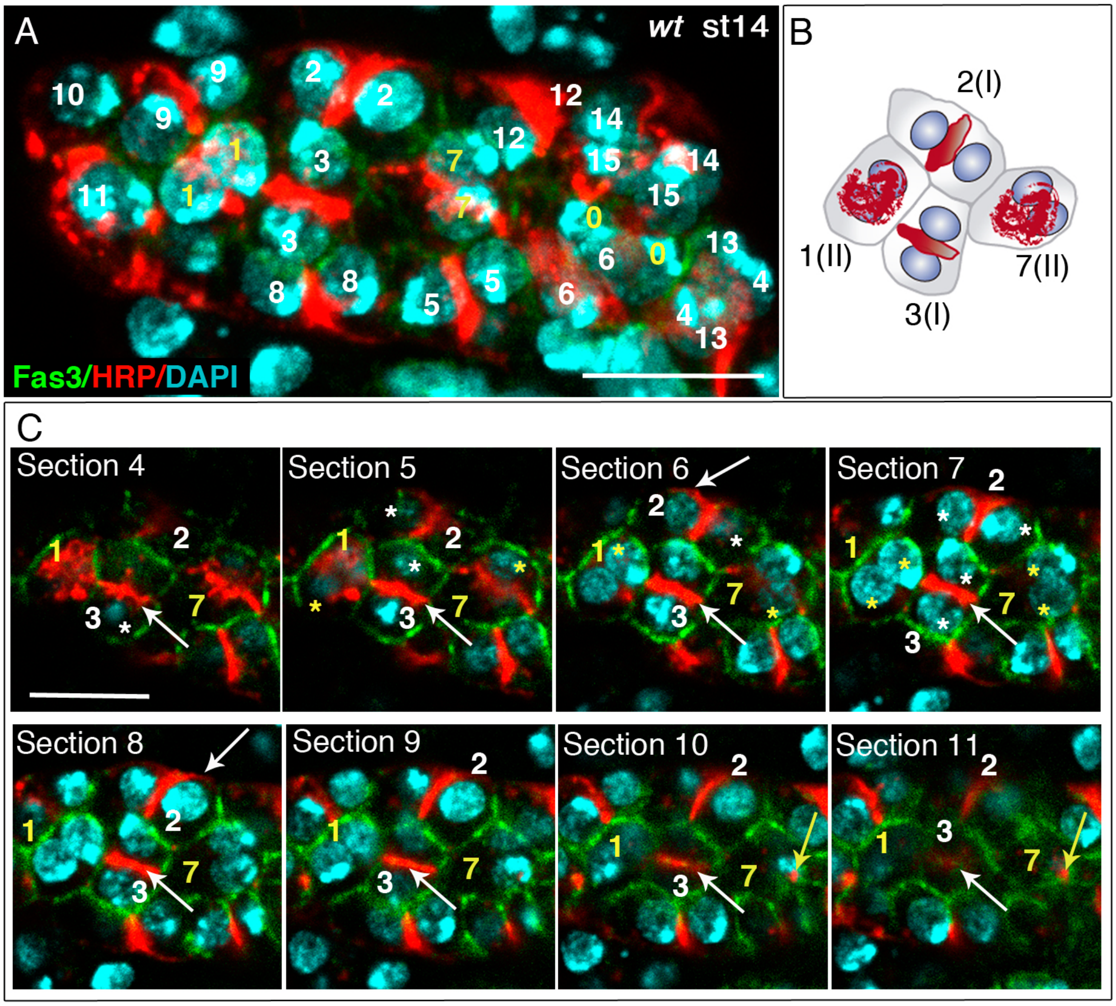

Fig. S2. Slit diaphragm distribution in garland nephrocytes at embryonic stage 14. (A-C) Confocal Z-projection (A), schematic (B) and consecutive single confocal sections (C) of a representative string of wild-type garland nephrocytes at embryonic stage 14 stained with anti-Fas3 and anti-HRP (n = 114 nephrocytes/ 8 independent strings); cell nuclei were counterstained with DAPI. The preferential accumulation of the HRP antigen at the equatorial wedges or forming an external cap allows the identification of two types of nephrocytes, type I and II, respectively. In panel A, both nuclei of each nephrocyte are marked with the number assigned to each cell, the white and yellow colours are used for nephrocytes type I and II, respectively. Both types are schematically represented for nephrocytes 1, 2, 3 and 7 in panel B. (C) The numbers indicate the different nephrocytes and the corresponding nuclei are labelled with asterisks. Note accumulation of HRP signal forming an external cap in type II-cell 1 (section 4) and the closely apposed nuclei below (sections 6-10), and the equatorial wedge separating sibling nuclei in type I-cell 2 (sections 4-11). The white arrows indicate a stronger accumulation of HRP at the external-most part of the equatorial wedge in sections 6 and 8 for cell 2, and 4-9 for cell 3 (compare with sections 10 and 11 in the latter). Similarly, in some instances nephrocytes with slit diaphragms concentrated in external caps present superficial membrane ingressions at the bottom of the cell (yellow arrows, basal plane of type II-cell 7 in sections 10-11). Scale bars represent 10 µm.

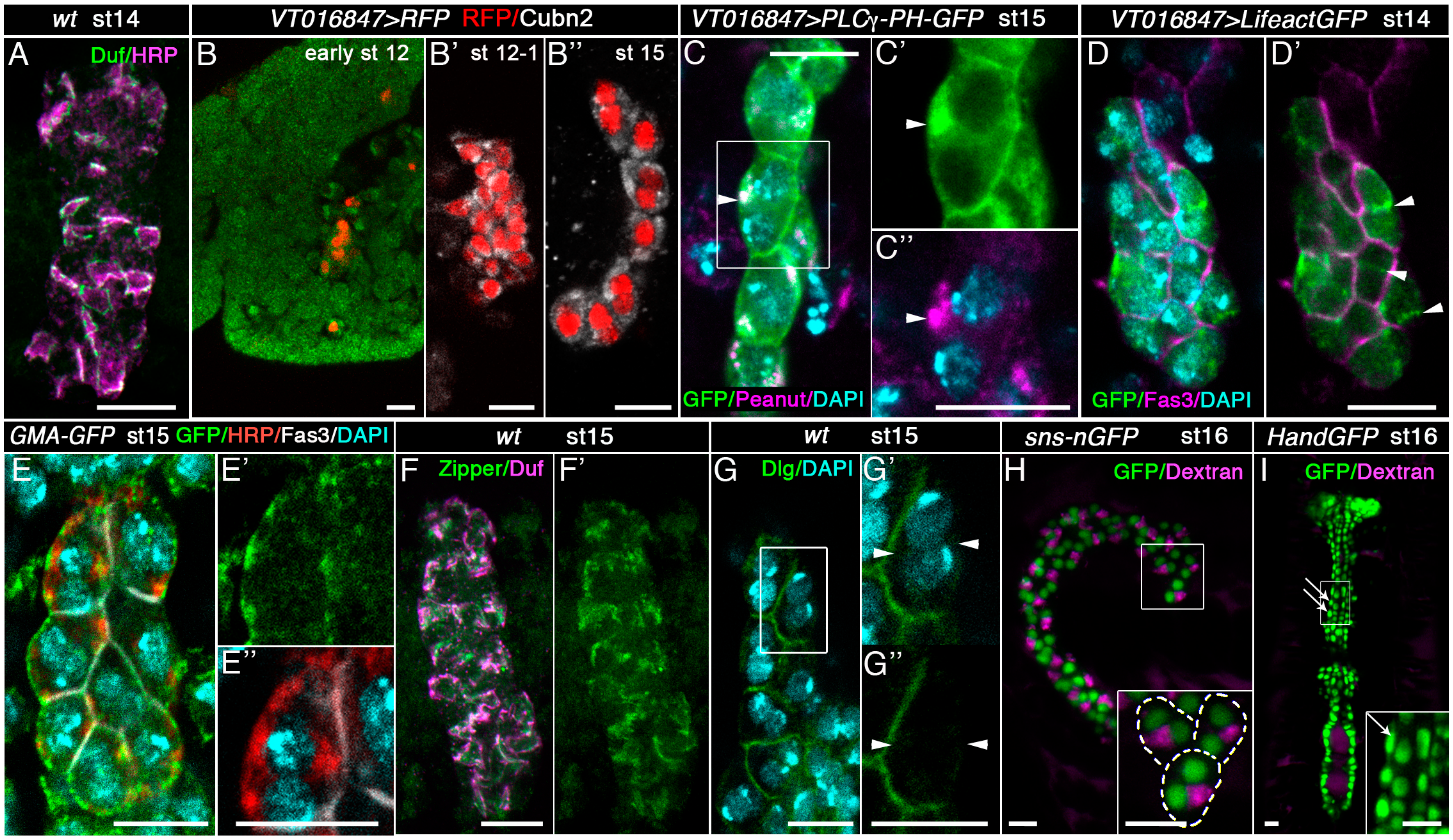

Fig. S3. VT016847Gal4 characterisation, subcellular localisation of selected markers and functional assays, in embryonic garland nephrocytes. (A-I) Confocal Z-projections (A-B" and F-I) and single sections (C-C", E-E') of embryonic garland nephrocytes of the indicated genotypes, stained as shown in the panels. (A) Glycoproteins recognised by anti-HRP staining accumulate at the equatorial wedges, and partially co-localise with the slit diaphragm proteins as shown for Duf (n = 55N/4S). (B-B") *VT016847Gal4* drives expression in embryonic nephrocytes (marked by Cubn2 accumulation) from stage 12. (C-C") Peanut co-localisation with PIP2 at the equatorial region of nephrocytes (white arrow, n = 25N/5S). (D-D') F-actin (labelled by *Lifeact-GFP*) accumulates in the equatorial region of stage 14 nephrocytes (arrowheads, n = 8N/2S). Cell contours are labelled by Fas3 accumulation. (E-F') Distribution of F-actin (marked by *GMA-GFP,* E, E') and Zipper (F, F') at the equator of stage 15 nephrocytes (n = 26N/3S and n = 36N/3S respectively). (G, G') Dlg accumulates at the membrane contacting adjacent nephrocytes (arrowheads in G', G" indicate the equatorial plane, n = 36N/3S). (H, I) Embryonic garland nephrocytes, recognised by expression of GFP in *sns-nGFP* (H and inset, n = 8/10 injected embryos), but not pericardial nephrocytes (expressing GFP in the line *Hand*-GFP, arrows in I and inset, n = 9/9 injected embryos) can uptake dextrans from the haemolymph in embryonic stages. Scale bars represent 10 μm.

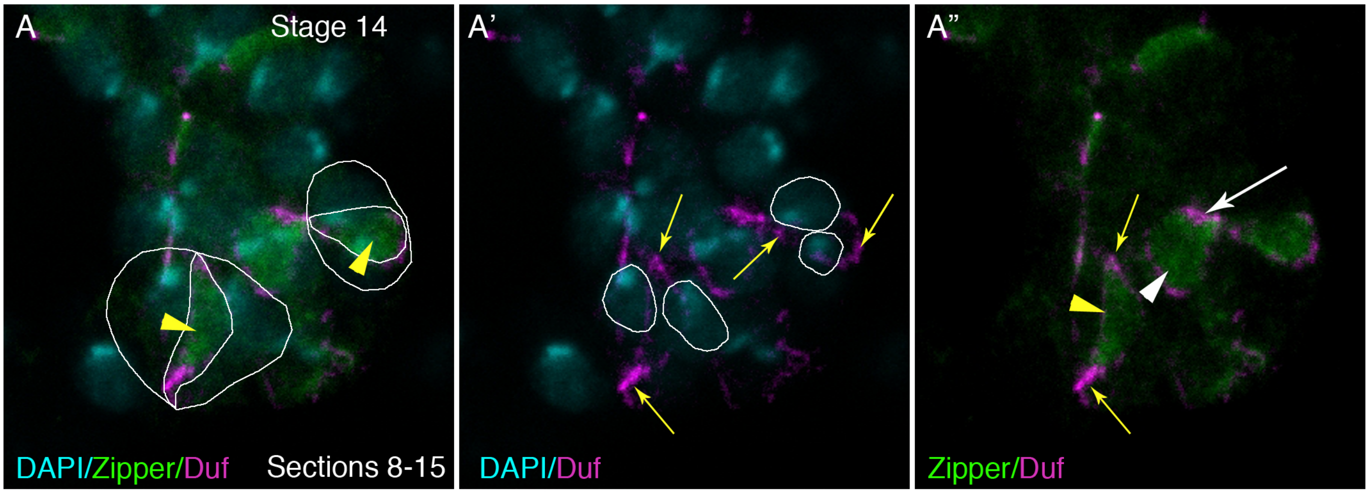

Fig. S4 (related to Fig. 3H). Accumulation of Zipper and Duf in stage 14 *wild-type* garland nephrocytes. (A-A") Detail of a Z projection of sections 8 to 15 of the string of nephrocytes from Fig. 3H, to show the accumulation of Zipper and Duf in relation to the position of the sibling nuclei. (A) Cell contours and equatorial wedges (accumulating Zipper, arrowheads) are outlined for two cells. (A') The pairs of sibling nuclei of these cells are highlighted; arrows point to Duf accumulation at the equatorial cortexes. (A") Equatorial wedges and cortexes are marked by arrowheads and arrows, respectively. White symbols point to the cell similarly marked in Fig. 3H-H".

Development • Supplementary information

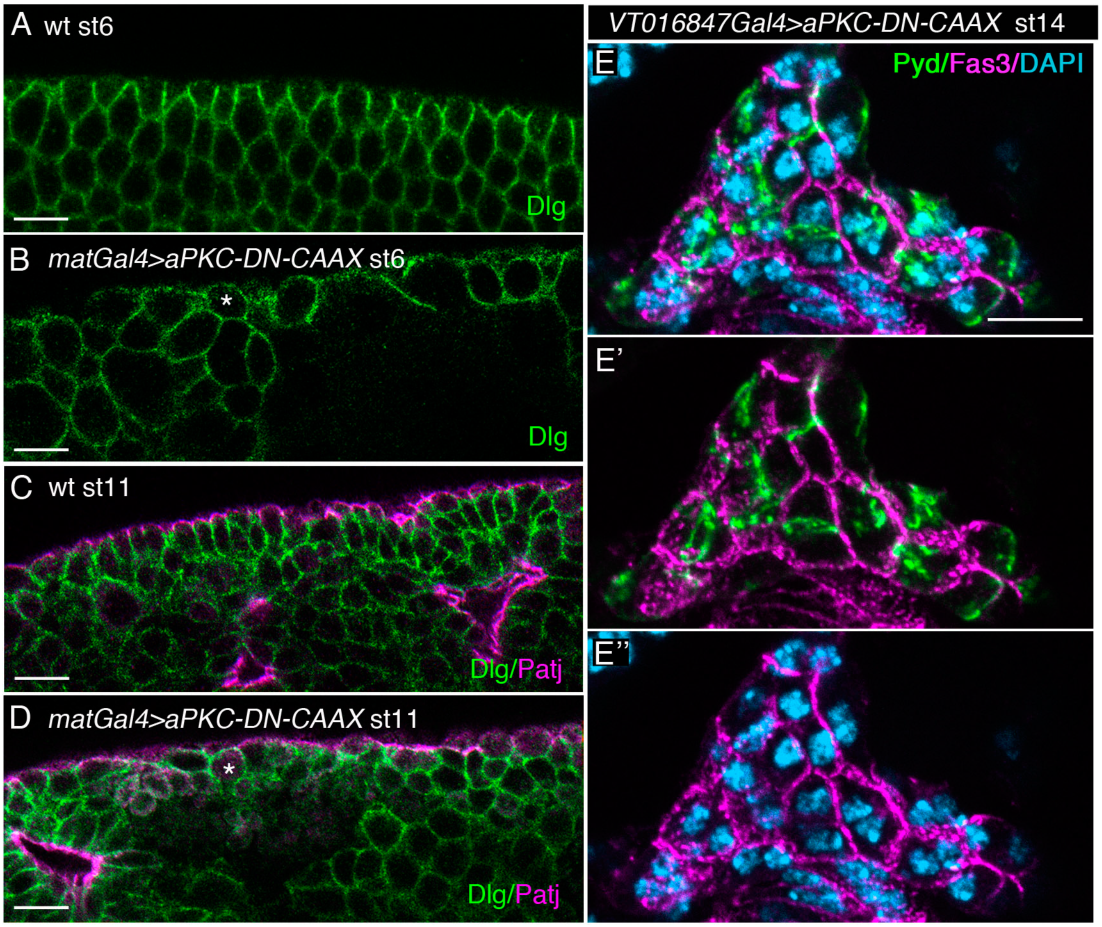

Fig. S5. Effect of the overexpression of DaPKC^{CAAXDN} in the embryonic epithelia and in garland nephrocytes. (A-D) Confocal single sections, showing the epidermis of embryos of the indicated genotypes and stages, stained with antibodies against Dlg (green) and Patj (magenta). In wild-type embryos (A, C), Dlg is localised at the basolateral membrane, whereas Patj marks the apical membrane. Embryos expressing *UAS-aPKC$^{CAAX\text{-}DN}$* with the maternal driver *matVP16V67-Gal4* (B, D) present an aberrant organization of the epithelia with a redistribution of Dlg to the whole membrane of some cells (asterisks). (E-E”) Z-projections of confocal images of st14 embryonic garland nephrocytes overexpressing *UAS-aPKC$^{CAAX\text{-}DN}$* driven by *VT016847-Gal4*, showing the normal localisation of Fas3 (magenta) and Pyd (green), which accumulates at the equatorial cortex, in binucleated nephrocytes. Compare with the distribution of Fas3 and Pyd in wild-type nephrocytes (Fig. 1C-C’”). Blue corresponds to DAPI staining in E. Scale bars: 10 µm.

Development • Supplementary information

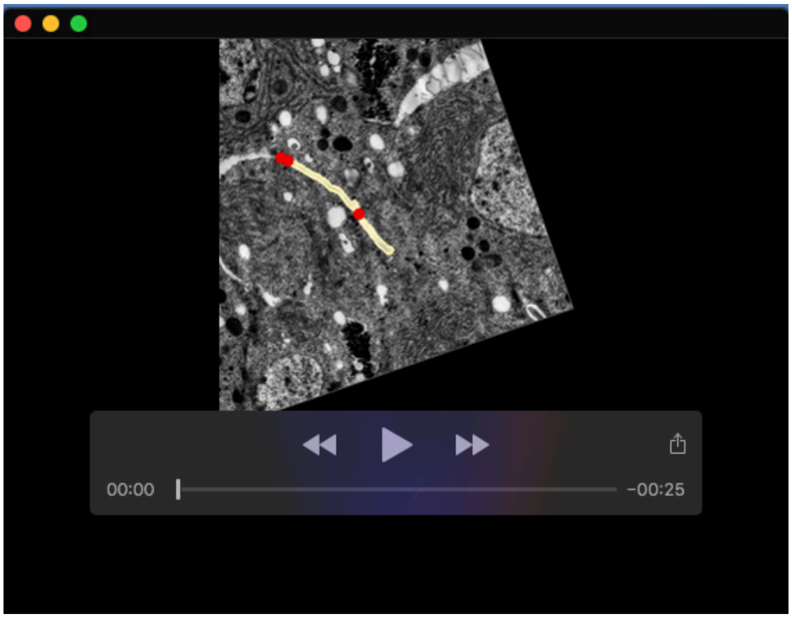

Movie 1. Serial sections of a nephrocyte cut perpendicularly to the equatorial plane. Related to Fig. 2D**.** Segmentation of aligned serial sections obtained for cell 1, showing slit diaphragms in red, lacunae in yellow and nuclei in blue.

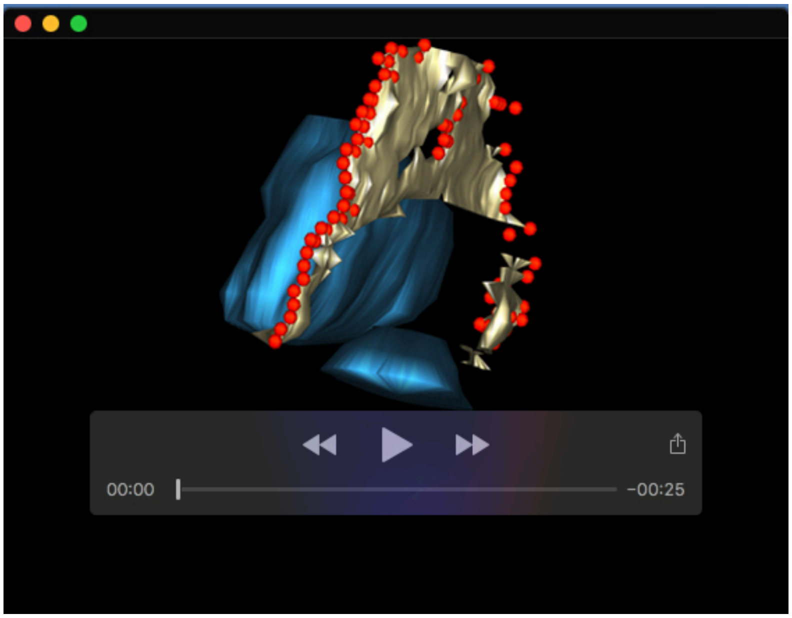

Movie 2. 3D reconstruction of the segmentation for cell 1 displayed in movie 1. Related to Fig. 2F,F'. 3D reconstruction of the segmentation showed in Movie 1 using 3dmod GUI. (graphical user interface).

Development • Supplementary information

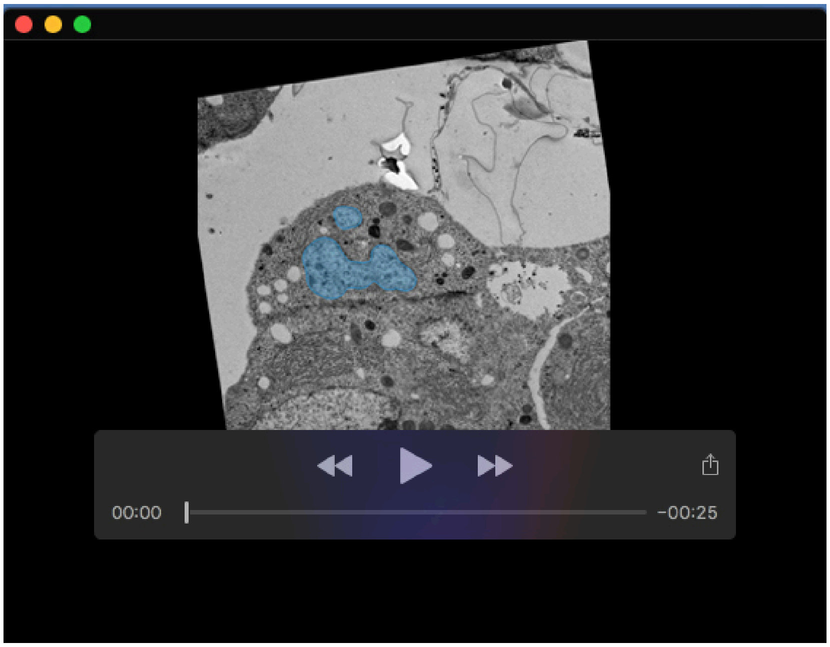

Movie 3. Serial sections of a nephrocyte cut through the equatorial plane.
Related to Fig. 2E. Segmentation of aligned serial sections obtained for cell 2, showing slit diaphragms in red, lacunae in yellow and nuclei in blue.

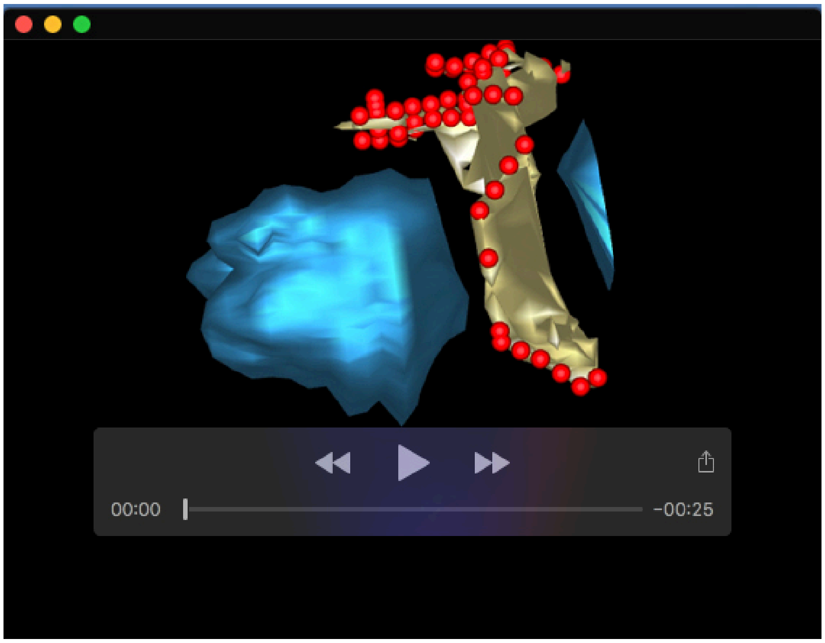

Movie 4. 3D reconstruction of the segmentation for cell 2 displayed in Movie 3. Related to Fig. 2G,G'. 3D reconstruction of the segmentation showed in Movie 3 using 3dmod GUI.

Development • Supplementary information
