# Supplementary material for: An acytokinetic cell division creates PIP2-enriched membrane asymmetries leading to slit diaphragm assembly in Drosophila nephrocytes
Source: Development. 2023 Sep 18;150(18):dev201708. doi: 10.1242/dev.201708 (PMC10546876; doi:10.1242/dev.201708)
Supplement: Supplementary file 1 [file develop-150-201708_review_history.pdf]

An acytokinetic cell division creates PIP2-enriched membrane asymmetries leading to slit diaphragm assembly in Drosophila nephrocytes

Marta Carrasco-Rando, Joaquim Culi, Sonsoles Campuzano and Mar Ruiz-Gómez
DOI: 10.1242/dev.201708

Editor: Thomas Lecuit

Review timeline

Original submission:	16 February 2023
Editorial decision:	24 March 2023
Rebuttal submitted:	29 March 2023
Editorial decision:	3 May 2023
First revision:	26 July 2023
Accept:	31 August 2023

Original submission

First decision letter

MS ID#: DEVELOP/2023/201708

MS TITLE: An acytokinetic division creates PIP2-rich membrane asymmetries leading to slit diaphragm assembly in Drosophila nephrocytes

AUTHORS: Marta Carrasco-Rando, Joaquim Culi, Sonsoles Campuzano, and Mar Ruiz-Gómez

I have now received all the referees' reports on the above manuscript, and have reached a decision. I am very sorry to say that the outcome is not a positive one. The referees' comments are appended below, or you can access them online: please go to Development's submission site and click on the 'Manuscripts with Decisions' queue in the Author Area.

As you will see, two of the referees raise some significant concerns about your paper, and are not strongly in favour of publication. They both appreciate the interest of this system, and value the quality of the data reported, yet they argue that the data are too preliminary and descriptive and therefore the dissection of the underlying mechanisms is insufficient. Having looked at the manuscript myself, I tend to agree with their views, and I must therefore, reject your paper.

I do realise this is disappointing news, but Development receives many more papers than we can publish, and we can only accept manuscripts that receive strong support from referees.

I do hope you find the comments of the referees helpful, and that this decision will not dissuade you from considering Development for publication of your future work. Many thanks for sending your manuscript to Development.

© 2023. Published by The Company of Biologists under the terms of the Creative Commons Attribution License (https://creativecommons.org/licenses/by/4.0/).

Reviewer 1

Advance summary and potential significance to field

Carrasco-Rando and colleagues describe how Drosophila embryo nephrocytes organize their plasma membrane domain structure and cell shape for their function in haemolymph filtration. The cells are shown to be bi-nucleated, resulting from incomplete cell division rather than cell-cell fusion. The incomplete cell division is followed by specialization of the pseudocleavage furrow through the recruitment of slit diaphragm adhesion/filtration proteins to its perimeter resulting in junctional complexes detected by TEM between opposing membranes of the furrow. Later, these proteins and the associated domain expand their distributions. The authors focus on the initial formation of the domain. PIP2 localizes across the domain, as do apical membrane markers and components of the cytokinetic ring. Preventing cell cycle progression or forcing complete cell division both disrupt development of the domain, whereas expanding the distribution of the apical protein aPKC does not prevent formation of the domain but does have other effects, confirming ectopic aPKC activity. The authors suggest that "membrane domains enriched in PIP2 could facilitate the concentration and/or organisation of slit diaphragm components such as Sns and Duf (both resident in lipid raft microdomains) and thus, trigger slit diaphragm assembly." The combination of detailed and high-quality confocal and TEM microscopy is a strength of the paper, together with the application of genetic perturbations. The main findings are of interest, but a number of issues should be addressed.

Comments for the author

1. The authors make many strong claims about the role of PIP2 in organizing the domain, including the title of the paper, but there are two major concerns.
1a. It is not conclusively shown that PIP2 is enriched within the membrane of the pseudocleavage furrow. It remains possible that the elevated signal is the result of the additive effect of two closely apposed membranes that can't be resolved by light microscopy. To distinguish these possibilities, quantitative comparisons should be made with controls, e.g. different domains of the PM with cell-cell contacts and/or imaging of distinct general PM markers.
1b. PIP2 levels are not experimentally decreased to test if PIP2 is required for domain organization.
2. The authors state that "pyd-deficient embryos…do not form slit diaphragms (Figures 4A-A')". A magnified view should be added to make this clear, as done elsewhere in the figures.
3. The authors show that Sqh[DD] over-expression results in mono-nucleated nephrocytes in larvae, and that the slit diaphragm is normal at this stage presumably because of compensatory mechanisms, but they don't analyze the effect of this over-expression in the embryo, as they do for overexpression of Pebble.
Thus, the Sqh[DD] over-expression analysis isn't conclusive, and should be expanded to match the scope of the Pebble overexpression analyses.
4. In Figure 4K and K', the localization of Pyd in relation to Fas3 is unclear.
Pyd should be shown as a single channel image, as done for Fas3.
5. The authors state, "Overexpression of DaPKCCAAX, and DaPKCCAAXDN did not overtly modify the localisation of slit diaphragm proteins, nor the timing of the binuclation process." It is not clear that the over-expression of these proteins had any effect. If a positive control for an effect can't be provided, then the statement should be removed.
6. The authors should be cautious about suggesting involvement of membrane rafts.
No data is provided that directly supports raft involvement.

Smaller issues:
-In Figure 1, the same colors should be used to show Duf and Pyd throughout the figure (D and D' are different than the rest).
-Using red with green in dual channel images is problematic for people who are colour blind.
-Typo on line 135: "aPCK"
-Typo on line 369: "wilt-type"
-The authors should be cautious of interpreting phosphorylated aPKC as active unless they can provide a reference showing that this is the case in Drosophila.

© 2023. Published by The Company of Biologists under the terms of the Creative Commons Attribution License (https://creativecommons.org/licenses/by/4.0/).

Reviewer 2

Advance summary and potential significance to field

Slit diaphragms are specialized cell junctions that perform blood filtration in the kidney as part of excretion. These structures are conserved in specialized kidney cells termed nephrocytes that perform the same function by filtering hemolymph in Drosophila melanogaster embryos. The current manuscript addresses the question of how slit diaphragms form in nephrocytes. In particular, they examine the initial stages of slit diaphragm formation in a group of embryonic nephrocytes called garland cells.

Developing garland cells undergo an extra postblastoderm division involving karyokinesis in the absence of cytokinesis, which leads the cells to become binucleate. In examining these cells, the authors discover the existence of a wedge of ingressed membrane that forms in the equatorial region between the two nuclei, accumulates the membrane phospholipid PIP2 (which they show colocalizes with slit diaphragm proteins), and serves as the initial site of slit diaphragm formation. In addition to PIP2, they find that myosin II accumulates throughout the equatorial wedge and the septin Pnut has patchy accumulation at the equatorial cell cortex. They note that the distribution of neither myosin II nor Pnut appears to change during this process, suggesting that a contractile ring does not form and constrict. They hypothesize that this membrane forms as a consequence of an incomplete cytokinesis event, which they term “acytokinetic cell division”, and they perform a series of experiments aimed at testing whether this is indeed the case. For example, they perturb cytokinesis using CycA mutant embryos, which produce mononucleated nephrocytes, and overexpression of the RhoGEF Pebble or constitutively active myosin regulatory light chain (Sqh), which also produce mononucleated nephrocytes, albeit with low penetrance. These perturbations delay the formation of slit diaphragms, consistent with the authors’ claim that acytokinetic cell division might be involved. In addition the authors largely rule out a role for apical polarity determinants such as aPKC in regulating slit diaphragm formation in these cells.

Overall, the manuscript provides clarity regarding a series of events that occur in garland cells that permit them to form slit diaphragms at an early stage of embryonic development. The authors have done an excellent job of describing this in fixed samples at the level of membrane lipid and protein localization as well as ultrastructural detail.

Comments for the author

The manuscript as it stands is largely descriptive, as the experimental results do not provide a satisfying mechanism to explain how formation of the membrane ingression that leads to assembly of the initial slit diaphragms might occur. For example, it is unclear what drives the initial polarization of the various components to the equator, nor what induces formation of the ingressed membrane wedge. Mechanistically, it seems important to know whether microtubules involved in karyokinesis direct Rho, anillin, F-actin, etc. to the cell equator to promote initial formation of the membrane wedge (do these proteins initially accumulate at the equator, and do they form a ring that at least partially constricts?) or if factors involved in secretion, endocytosis, recycling (for example, of slit diaphragm proteins) or membrane curvature (BAR domain proteins, etc.) are involved in promoting membrane ingression and slit diaphragm assembly.
If the authors are to revise the current manuscript for submission to Development or another journal, the following major points should be addressed:
1. In the Summary Statement, the authors refer to a “stalled cell division (without cytokinesis)”, yet their data do not strongly support this, as there is no clear evidence of initiation of the last stage of cell division (i.e., assembly of an equatorial contractile ring during cytokinesis) as the initiating step in formation of the equatorial wedge that leads to slit diaphragm formation (see above comments). If this is not the proposed model, it would help for the authors to more precisely define what they mean by “stalled cell division” and “acytokinetic cell division” so as not to confuse the reader.

2. The suggestion that “the site of slit diaphragm proteins accumulation, at the equator of stage 14 nephrocytes, might be marked by prior positioning of the cytokinetic ring” (lines 184-185) is never

© 2023. Published by The Company of Biologists under the terms of the Creative Commons Attribution License (https://creativecommons.org/licenses/by/4.0/).

fully substantiated by the data (neither F-actin nor anillin are shown to be present, for example) and is therefore a bit misleading. Softening this to “a cytokinetic ring” here and in subsequent sections (line 249, line 253, etc.) might be more reasonable in light of the data presented. Along these lines, live imaging of spindle microtubules and cytokinetic markers would be much more convincing with regard to the timing and establishment of a cytokinetic ring than the images provided.

3. It is not clear whether PIP2 appears to “exhibit a robust accumulation at the cell equator” (line 259; Fig. 3A’) due to the presence of the double layer of plasma membrane (as seen in the TEM images) or if it is enriched relative to the amount of membrane that is present (one would expect PIP2 to be twice as bright where there are two layers of membrane). To address this, it is important to examine a non-specific plasma membrane marker (e.g., FM1-43 or a myristoylated fluorescent protein, etc.) to see if membrane in general (not just PIP2) appears similarly enriched in this location, as this would change the interpretation of the results with the PIP2 marker and might require the authors to change the title of the manuscript.

ADDITIONAL MINOR COMMENTS

Minor points in the text:
1. Missing word “on” after “confer” (line 59).
2. Define sns-GCN-nGFP when first mentioned (line 164).
3. “Effectively” (line 176) is unclear and could be deleted, and the sentence (esp. line 177) should be rewritten for clarity.
4. Similarly, at the bottom of the page, it would help to start new sentence with “In contrast,” instead of “whereas” (line 180) for clarity.
5. “by large” is confusing (lines 218-219) and could be deleted.
6. “Noteworthy” (line 273) could be replaced by “Of note,”.
7. For clarity, move “PIP2” (line 277) to the next line, before “would”.
8. Fix typos: “slit” (line 343) and “wild-type” (line 369).

Minor comments on the figures:
1. Colored labels on the gray boxes above the panels (Figs 1, 3, 4, S1, S3) are difficult to read, especially when printed out.
2. In general, it would help to show single color images (Fig. 1B’, F’’’, G’’, I) in grayscale rather than color, as grayscale is easier to see on a black background. Also, for two color images, magenta and green is better than red and green for colorblind readers.
3. Some of the arrowheads are extremely small and difficult to see when printed (for example, in Fig. 1B, B’). Similarly, some of the arrows are extremely skinny and hard to see (Fig. 1C-C’’, D-D’, E, K’, L’). The blue asterisk (Fig. 1L’, upper inset) is nearly impossible to see.

Minor comments on the supplementary figure legends:
1. The interpretation in the last sentence of the Fig. S1 legend, “indicating that binucleation of nephrocytes is the result of cell divisions” (lines 15-16) seems an overstatement; suggest replacing “is the result of” with “correlates with”.
2. It’s unclear what is meant by “just opposite to the outer membrane” (line 36) in the Fig. S2 legend.

<u>Reviewer 3</u>

Advance summary and potential significance to field

In their manuscript, Carrasco-Rando et al. describe the process of slit diaphragm formation in the development of Drosophila nephrocytes, cells, which show similarity with mammalian podocytes. The authors convincingly show that incomplete cytokinesis generates the first membrane asymmetry with local accumulation of PI(4,5)P2 and slit diaphragm components at this site. Forced completion of cytokinesis delays the formation of slit diaphragms.

© 2023. Published by The Company of Biologists under the terms of the Creative Commons Attribution License (https://creativecommons.org/licenses/by/4.0/).

The manuscript is well written and follows a comprehensible red line. The experiments mostly support the conclusions and the findings are of great value for our understanding of slit diaphragm development.

Comments for the author

However, I have several points, which should be addressed prior to publication:

Fig. 1A: Why are Pyd and Duf not clustered at all tricellular junctions? Some spots are clearly outside tricellular junctions, although it is hard to judge in this resolution. Thus, a co-staining with a marker of tricellular junctions (sidekick) would help here – staining of sidekick (Fig. 1B) alone is dispensable in this context.

Fig. 3: Intensities of PLC-PH-GFP accumulation at cytokinesis furrows in contrast to other membranes should be quantified. A slit diaphragm marker (Pyd/Duf) as well as stainings against the Septin Peanut should be used together with PLC-PH-GFP to verify that slit diaphragm proteins accumulate at sites with high PIP2 density at the cytokinesis ring.

Fig. 3D: DAPI staining (cp Fig. 3B''') would help to identify binucleated nephrocytes Fig. 4: Are slit diaphragms recovered at later stages (e.g. in larval stages), if acytokinetic cell division is blocked (maybe here a guide or shRNA line against CycA instead of cycA mutants must be used)?

Fig. 4J: The number of slit diaphragms formed in Pebble/Sqh-overexpressing nephrocytes should be quantified to demonstrate that nephrocytes, which complete cytokinesis exhibit no defects in slit diaphragm formation in larval stages.

Fig. 4K: The control situation of nephrocytes at stage 16 should be shown (as in Fig. 1E) for better comparison)

Overexpression of Pbl in otherwise wild type nephrocytes is capable to induce a completion of cytokinesis with low penetrance. Why is the accumulation of slit diaphragms incomplete/delayed in all cells (mono- and binucleated cells) at stage 16, which hints at a role of Pbl/Myosin contraction in positioning/stabilizing slit diaphragms independently of the cleavage furrow?

Line 350ff: In my eyes, the explanation of the recruitment of additional slit diaphragms is not logic: The first strand of slit diaphragms assemble at the cleavage furrow due to incomplete cytokinesis, which was nicely demonstrated – how should these complexes recruit the next strand, approximately 300-400nm apart from the first strand?

Fig. 4L: I suggest to take out the data of apical polarity regulators (aPKC) affecting early slit diaphragm formation, otherwise more experiments are needed to address this (interesting) question, e.g. by downregulation/mutation of aPKC and Baz (which directly binds to PI(4,5)P2). In the current version, the results that overexpression of an activated version of aPKC delays acytokinetic division and slit diaphragm formation remains unexplained (or is explained by functions of aPKC which are independent of its role in polarity) and does not contribute to the main findings of the manuscript.

Finally, in the title the authors suggest a causative role for PI(4,5)P2 leading to slit diaphragm assembly. While this is quite likely and suggested by another study (Gass et al. 2022), too, the shown experiments do not (yet) support this conclusion. Downregulation of PI(4,5)P2 in developing nephrocytes should block accumulation of slit diaphragm components at the cytokinesis furrow and demonstrate a causative role of this phospholipid as suggested by the authors.

Minor points

Fig. 1C – A DAPI staining labelling the nuclei (as shown in C'') would help to distinguish single binucleated nephrocytes.

© 2023. Published by The Company of Biologists under the terms of the Creative Commons Attribution License (https://creativecommons.org/licenses/by/4.0/).

Fig. 2: Quantifcation of nephrocyte cell types should be displayed in the main figure, not in the supplements Line 329: It should become clear, that “experimental nephrocytes” are nephrocytes with an overexpression of Pbl.
Line 369: Typo: wild-type Line 343: Typo: slit diphragms

Rebuttal letter

Dear Thomas Lecuit,

It is very disappointed news indeed. However, I would like to take the opportunity of you being an active researcher in *Drosophila* cell biology to argue the reasons why we think our work will be of interest for a wide audience of “Development” readers, including cell and developmental biology researchers and nephrologists. I hope that my arguments will move you to reconsider your decision and give us the opportunity to submit a revised version of our manuscript to “Development”.

A pending question in Nephrology is unravelling the mechanisms underlying the transition from occludens junctions to slit diaphragms, which takes place during the early stages of vertebrate podocyte development. In *Drosophila,* the generation of slit diaphragms takes place in cells of mesodermal origin, lacking any overt apicobasal polarity, and therefore it is very relevant to understand how symmetry is broken in these cells to generate membrane domains prone to harbour slit diaphragm proteins. Our results indicate for the first time that binucleated nephrocytes don´t arise from cell fusion events, as it was assumed, but from incomplete cytokinesis. They also show that this incomplete cytokinesis of stage 13 embryonic nephrocytes allows the formation of an equatorial wedge initiated by the assembly of a cytokinetic ring, enriched in PIP2, which creates a favourable environment for the clustering of slit diaphragm proteins.

We also experimentally prove that the prolonged maintenance of this domain is relevant for facilitating slit diaphragm assembly. Thus, in *CycA* mutants, where such division does not take place and there is a failure in the formation of such membrane domain, or when cytokinesis is forced to its completion (by overexpression of active *sqh* or *pebble*), the assembly of slit diaphragm is delayed until larval stages. Although this mechanism to account for the origin of the membrane asymmetry that sustains slit diaphragm assembly might apply only to nephrocytes, it reveals the relevance that PIP2 accumulation has in creating a specialised membrane domain that may be operative during podocyte maturation in vertebrates. In this regard, we have also addressed an issue that has not been resolved in podocytes, the possible role played by apicobasal determinants in the assembly of slit diaphragms, and we propose a hypothesis, supported by solid experimentation, about the mechanisms involved in the formation of the slit diaphragms in *Drosophila* embryonic nephrocytes.

The three reviewers show a common concern regarding the role of PIP2 in organising the above-mentioned membrane domain. They say that “*it has not been conclusively shown that PIP2 is enriched within the equatorial nephrocyte membrane, since the elevated signal may be the result of the additive effect of two closely apposed membranes*”. This query can be experimentally solved by looking, as suggested, at the accumulation of PM markers at this membrane domain. In addition, we can also look at PIP2 accumulation at the equatorial membrane in *pyd* mutants. In this mutant, apical and slit diaphragm proteins (other than Pyd) still accumulate at the equatorial membrane but due to the absence of Pyd slit diaphragms do not form and therefore nephrocytes lack membrane ingressions. If accumulation of PIP2 is still detected at the equatorial wedge in this mutant background, it cannot be attributable to apposed membranes.

In addition, the reviewers also mention that “*PIP2 levels are not experimentally decreased to test if PIP2 is required for domain organization*”. We agree with them that showing an effect of manipulation of PIP2 levels on embryonic nephrocytes should strength our hypothesis. This approach, although experimentally challenging, could be addressed. as well as other suggestions and comments of the reviewers. Reviewer 2 points out that “*the experimental results do not provide a satisfying mechanism to explain: a) “what drives the initial polarization of the various components to the equator” and b) “what induces formation of the ingressed membrane wedge*”.

© 2023. Published by The Company of Biologists under the terms of the Creative Commons Attribution License (https://creativecommons.org/licenses/by/4.0/).

However (a), we have provided a mechanism to explain the initial polarization of several proteins (slit diaphragm components and polarity determinants) to the equator as a result of the assembly of a cytokinetic ring, whose existence is revealed by the accumulation of the motor protein MyoII and the septin Peanut, and results in the accumulation of PIP2 at this equatorial domain, which generates a suitable environment for the above mentioned proteins to accumulate at it. Certainly, we could have used more tools to describe the assembly of the cytokinetic ring, but this is not the scope of the article. Furthermore, a functional proof of this assembly is that by activating MyoII or supplying Pebble we can force the constriction of this cytokinetic ring, causing a completion of cytokinesis. Regarding the formation of the ingressed membrane wedge (b), we hypothesise it is driven after the assembly of the slit diaphragms, sealing the initial ingression at the equatorial edge, by mechanisms of membrane remodelling (involving endo and exocytosis and recycling) similar to the ones previously described by us and other groups for the enlargement of the labyrinthine channels. This interpretation is supported by the observation that in *pyd* mutant embryos there is no membrane ingression despite the generation of the equatorial membrane asymmetry that allows accumulation of both slit diaphragm components and polarity determinants at this location (Figure 4).

Despite the reviewer's criticism, they find our work relevant and appropriate for the journal: "*The combination of detailed and high-quality confocal and TEM microscopy is a strength of the paper, together with the application of genetic perturbations. The main findings are of interest, but a number of issues should be addressed*" (Reviewer 1), "*Overall, the manuscript provides clarity regarding a series of events that occur in garland cells that permit them to form slit diaphragms at an early stage of embryonic development. The authors have done an excellent job of describing this in fixed samples at the level of membrane lipid and protein localization as well as ultrastructural detail.*" (Reviewer 2) and "*The manuscript is well written and follows a comprehensible red line. The experiments mostly support the conclusions and the findings are of great value for our understanding of slit diaphragm development.*" (Reviewer 3)

For these reasons, we ask you to reconsider your decision, and allow us to submit a revised version of the manuscript to Development where we will be willing to address all concerns raised by the reviewers.

Rebuttal decision letter

MS ID#: DEVELOP/2023/201708
MS TITLE: An acytokinetic division creates PIP2-rich membrane asymmetries leading to slit diaphragm assembly in Drosophila nephrocytes
AUTHORS: Marta Carrasco-Rando, Joaquim Culi, Sonsoles Campuzano, and Mar Ruiz-Gómez

Dear Dr. Ruiz-Gómez,

Thank you for your appeal on your recently rejected manuscript. I do understand your disappointment, but given the opinions expressed by the reviewers, I saw little option other than to decline the paper.

However, we are always willing to give authors the chance to defend their manuscripts, and I do recognise that you make some valid comments in your letter. Therefore we would be willing to reconsider a revised version of your manuscript that deals, as far as possible, with the points raised by the reviewers. Upon resubmission, please provide a detailed response to the reviewers' comments and highlighting particularly any concerns that have not been included in the revised manuscript.

The revised manuscript and rebuttal will be sent to the original reviewers (if they are still available). If they are convinced by your arguments, then we would be able to consider the manuscript for publication.

© 2023. Published by The Company of Biologists under the terms of the Creative Commons Attribution License (https://creativecommons.org/licenses/by/4.0/).

To submit a revision, please go to your Author Area and click on the 'Submit a Revision' link.

First revision

Author response to reviewers' comments

Detailed list of responses to the reviewers:
Reviewer #1

Major points:

The authors make many strong claims about the role of PIP2 in organizing the domain, including the title of the paper, but there are two major concerns.

Query1a. It is not conclusively shown that PIP2 is enriched within the membrane of the pseudocleavage furrow. It remains possible that the elevated signal is the result of the additive effect of two closely apposed membranes that can't be resolved by light microscopy. To distinguish these possibilities, quantitative comparisons should be made with controls, e.g. different domains of the PM with cell-cell contacts and/or imaging of distinct general PM markers.

Authors' response:
We thank all reviewers for pointing this out and understand the concern raised by them regarding PIP2 enrichment in the equatorial membrane. To clarify this question, we are presenting in the revised version of the manuscript additional data that substantially support our conclusion that such PIP2 enrichment is not due to the presence of the double layer of plasma membrane. We have quantified the intensity of PIP2 staining with the PIP2 sensor PLCg-PH- GFP at the equatorial wedge and at the site of cell-cell contacts in wild-type nephrocytes. Both sites contain two closely apposed membranes but as shown in Fig. 3A,B, signal intensity at the equator is double than that at regions of cell contacts. In addition, we have also included similar quantifications in *pyd*^{ex147} mutant nephrocytes, where equatorial membrane ingressions do not form, showing that PIP2 still accumulates twice at the equatorial cortex than at cell-cell contacts (Figs 3B, 4A,C-C').

Query 1b. PIP2 levels are not experimentally decreased to test if PIP2 is required for domain organization.

Authors' response:
Experimental approaches to decrease PIP2 levels in embryonic nephrocytes are not feasible due to technical limitations as now indicated in the text. Thus, optogenetic manipulation of PIP2 levels in embryonic nephrocytes is precluded by their location deep inside the embryo and floating in the haemolymph. We considered to modify PIP2 levels by manipulating the Inositol 5-phosphatase dOCRL, which desphosphorylates PIP2. However, analysis of the effect of dOCRL loss in nephrocytes was prevented by the persistent maternal activity in null *docrl*$^{\Delta 3}$ mutants, which survive until larval or pupal stages. In addition, RNAi-mediated attenuation is not possible, since it doesn't work efficiently during embryogenesis and in the case of *docrl*, the available RNAi lines have been reported to be insufficient to deplete *docrl*. Hence, we resorted to increase PIP2 levels in nephrocytes by overexpressing UAS-*docrl* with *VT016847-Gal4*. This experimental approach led to a premature accumulation of slit diaphragm proteins in the outer nephrocyte membrane, thus suggesting that PIP2 membrane enrichment directly affects the stabilisation of the diaphragm components in flies. These results are presented in the new Fig. 4D-D".

Query 2 The authors state that "pyd-deficient embryos…do not form slit diaphragms (Figures 4A-A')". A magnified view should be added to make this clear, as done elsewhere in the figures.

© 2023. Published by The Company of Biologists under the terms of the Creative Commons Attribution License (https://creativecommons.org/licenses/by/4.0/).

Authors' response:
The reason why a demonstration of the lack of SDs in pyd-deficient embryonic nephrocytes wasn't included in figure 4 is because this was already published in Carrasco-Rando et al., 2019. Nonetheless, following the reviewer's suggestion, a detail of the external membrane of the *pyd* nephrocyte shown in Fig. 4A, devoid of SDs, is now included in Fig. 4A".

Query 3. The authors show that Sqh[DD] over-expression results in mono-nucleated nephrocytes in larvae, and that the slit diaphragm is normal at this stage, presumably because of compensatory mechanisms, but they don't analyze the effect of this over-expression in the embryo, as they do for overexpression of Pebble. Thus, the Sqh[DD] over-expression analysis isn't conclusive, and should be expanded to match the scope of the Pebble overexpression analyses.

Authors' response:
Although not included in the previous version of the manuscript, we also analysed the embryonic effect of sqh^{DD} over-expression, which caused the same phenotype as *pbl* overexpression. We agree with the reviewer that without these data the sqh^{DD} over-expression analysis is incomplete, and therefore, in the revised version of the manuscript we have included data analysing the effect of sqh^{DD} over-expression in the embryo in the new Fig. 5F-F'".

Query 4. In Figure 4K and K', the localization of Pyd in relation to Fas3 is unclear. Pyd should be shown as a single channel image, as done for Fas3.

Authors' response:
Following the reviewer's suggestion, a single channel image showing Pyd localisation is now included in the new Fig. 5E', as done for Fas3 in Fig. 5E".

Query 5. The authors state, "Overexpression of DaPKCCAAX, and DaPKCCAAXDN did not overtly modify the localisation of slit diaphragm proteins, nor the timing of the binuclation process." It is not clear that the over-expression of these proteins had any effect. If a positive control for an effect can't be provided, then the statement should be removed.

Authors' response:
We agree with the reviewer and have modified the text accordingly. Since overexpression of constitutively active DaPKC-CAAX has not any detectable effect in embryonic nephrocytes, for simplicity, in the present version we have removed the data concerning DaPKC-CAAX overexpression. On the contrary, overexpression of constitutively active aPKC-DN, homogeneously distributed in the membrane, nuclei and cytoplasm of nephrocytes, did have an effect since it induced a delay in the acytokinetic division of the nephrocytes (shown in the new Fig. 5G-H'") although it does not affect the subcellular distribution of slit diaphragm proteins (new Fig. 5G-H'"). Regarding the activity of DaPKC-CAAX-DN, we have already shown that its overexpression interferes with the apical-basal polarity of embryonic ectodermal cells (Sotillos et al 2004, doi. 10.1083/jcb.200311031). As a positive control for the effect of the same line UAS-DaPKC-CAAX-DN that we have used now on nephrocytes, we provide the reviewer with an image showing the distorted apical-basal polarity of embryonic ectodermal cells resulting from its overexpression.

© 2023. Published by The Company of Biologists under the terms of the Creative Commons Attribution License (https://creativecommons.org/licenses/by/4.0/).

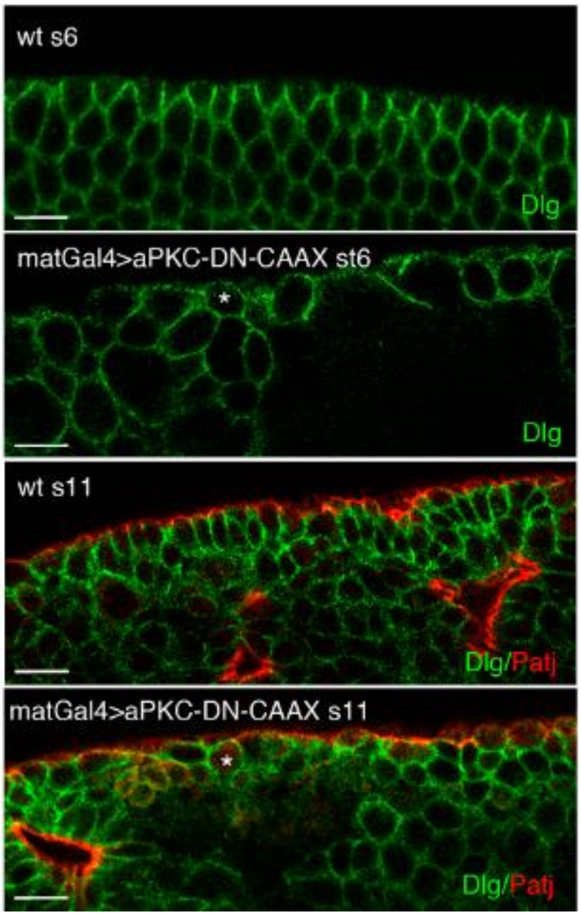

Confocal single sections, showing the epidermis of embryos of the indicated genotypes and stages, stained with antibodies against Dlg and Patj. In wild-type embryos, Dlg is localised at the basolateral membrane, whereas Patj marks the apical membrane. Embryos expressing *UAS-aPKC-CAAX-DN* with the maternal driver *matVP16V67-Gal4*, present an aberrant organization of the epithelia with a redistribution of Dlg to the whole membrane of some cells (asterisks).

Query 6. The authors should be cautious about suggesting involvement of membrane rafts. No data is provided that directly supports raft involvement.

<u>Authors' response:</u>
We thank the reviewer for this comment. Since in *Drosophila* the enrichment in lipid raft has only been demonstrated for the Duf paralogue Roughest (Rst; Hoehne et al., 2005, doi: 10.1016/j.mcn.2005.07.007), we have been very cautious throughout the manuscript and have avoided mentioning the association of *Drosophila* slit diaphragm proteins with lipid rafts. Note however, that it has been reported in several articles and it is generally accepted that the vertebrate slit diaphragm proteins nephrin and NEPH1 reside in lipid raft microdomains. Therefore, in the manuscript we mention only the association of vertebrate slit diaphragm components with lipid rafts.

<u>Smaller issues:</u>

1- In Figure 1, the same colors should be used to show Duf and Pyd throughout the figure (D and D' are different than the rest).

<u>Authors' response:</u>
We agree with the reviewer, and apologise for the mistake we made in the original figure. This is corrected in the new version of Fig. 1.

2- Using red with green in dual channel images is problematic for people who are colour blind.

© 2023. Published by The Company of Biologists under the terms of the Creative Commons Attribution License (https://creativecommons.org/licenses/by/4.0/).

Authors' response:

We thank the reviewer very much for this comment and have changed red for magenta in all images displaying double staining.

3- Typo on line 135: "aPCK"
4- Typo on line 369: "wilt-type"

Authors' response:
We thank the reviewer for pointing out these typographical errors that we have corrected in the revised version.

5- The authors should be cautious of interpreting phosphorylated aPKC as active, unless they can provide a reference showing that this is the case in Drosophila.

Authors' response:
We thank the reviewer for allowing us to clarify this point. In this report we are using anti p-Thr555-aPKCz (Abcam) as a readout of the presence of activated aPKC since it has been previously used for this purpose in at least two *Drosophila* studies: Wirtz-Peitz et al., 2008, doi: 10.1016/j.cell.2008.07.049 and Goh et al. 2013, doi: 10.1016/j.ydbio.2013.06.031.

Reviewer #2
Major points:
Query 1. In the Summary Statement, the authors refer to a "stalled cell division (without cytokinesis)", yet their data do not strongly support this, as there is no clear evidence of initiation of the last stage of cell division (i.e., assembly of an equatorial contractile ring during cytokinesis) as the initiating step in formation of the equatorial wedge that leads to slit diaphragm formation (see above comments). If this is not the proposed model, it would help for the authors to more precisely define what they mean by "stalled cell division" and "acytokinetic cell division" so as not to confuse the reader.

Authors' response:
We thank very much the reviewer for this constructive comment and have performed additional experiments to demonstrate the assembly of an actomyosin cytokinetic ring at the equatorial cortex of stage 14 garland nephrocytes. In the revised version of the manuscript we show the equatorial accumulation of Rho1 (Fig. 3C-C"), its downstream effector Dia (Fig. 3D-D'") and F-actin (Figs 3E-E", S3D,D'), in addition to myosin II/Zipper and Peanut shown previously (Fig. 3F-H'").

We are confident that the presence of these factors is indicative of the assembly of a cytokinetic ring and the initiation of the last stage of cell division, as it is the fact that the overexpression of Sqh^{DD} allows successful completion of cytokinesis (Fig. 5B).

Regarding the proposed model for slit diaphragm and equatorial wedge formation, what we suggest is that: (A) the accumulation of F-actin and myosin II filaments together with those of the actin crosslinking septin Peanut, which participates in the linking of the actomyosin ring with the cellular membrane, may lead to the assembly of actomyosin bundles at the equatorial plane, which could induce small ingressions of the membrane as a result of the sliding of actin filaments on myosin II that will shorten the bundles even in the absence of myosin contraction.

(B) these small membrane invaginations will be sealed by nascent slit diaphragms that assemble at the equatorial cortex rich in PIP2, where slit diaphragm proteins accumulate. (C) sealing of the edges of the invaginated membrane leads to the growth of the channels thus generated and to the formation of the equatorial wedges. This is supported by the observation that no equatorial wedges formed in the absence of slit diaphragms in pyd^{ex147} mutant nephrocytes, despite the accumulation of myosin, F-actin, PIP2 and slit diaphragm components at the equatorial cortex.

To explain it better, we have further developed this model in the discussion section of the revised manuscript.

© 2023. Published by The Company of Biologists under the terms of the Creative Commons Attribution License (https://creativecommons.org/licenses/by/4.0/).

To avoid confusions, we have also eliminated “stalled cell division” from the summary statement.

Query 2. The suggestion that “the site of slit diaphragm proteins accumulation, at the equator of stage 14 nephrocytes, might be marked by prior positioning of the cytokinetic ring” (lines 184-185) is never fully substantiated by the data (neither F-actin nor anillin are shown to be present, for example) and is therefore a bit misleading. Softening this to “a cytokinetic ring” here and in subsequent sections (line 249, line 253, etc.) might be more reasonable in light of the data presented. Along these lines, live imaging of spindle microtubules and cytokinetic markers would be much more convincing with regard to the timing and establishment of a cytokinetic ring than the images provided.

Authors’ response:
As mentioned above, we have included in the revised manuscript additional data that strongly support the assembly of a cytokinetic ring in stage 14 embryonic garland nephrocytes. However, since this additional post-blastoderm cell division lacks cytokinesis and is therefore atypical, following the reviewer’s suggestion, we have changed “the” cytokinetic ring to “a” cytokinetic ring throughout the manuscript.

We agree with the reviewer that live imaging of spindle microtubules would be ideal to show the assembly of a cytokinetic ring. However, one of the few limitations of working with embryonic garland nephrocytes is that they are located in an internal position, surrounding the oesophagus and floating in the haemolymph, which precludes live imaging or optogenetic experiments.

Query 3. It is not clear whether PIP2 appears to “exhibit a robust accumulation at the cell equator” (line 259; Fig. 3A’) due to the presence of the double layer of plasma membrane (as seen in the TEM images) or if it is enriched relative to the amount of membrane that is present (one would expect PIP2 to be twice as bright where there are two layers of membrane). To address this, it is important to examine a non-specific plasma membrane marker (e.g., FM1-43 or a myristoylated fluorescent protein, etc.) to see if membrane in general (not just PIP2) appears similarly enriched in this location, as this would change the interpretation of the results with the PIP2 marker and might require the authors to change the title of the manuscript.

Authors’ response:
We thank all reviewers for pointing this out and understand the concern raised by them regarding PIP2 enrichment in the equatorial membrane. To clarify this question, we are presenting in the revised version of the manuscript additional data that substantially support our conclusion that such PIP2 enrichment is not due to the presence of the double layer of plasma membrane. We have quantified the intensity of PIP2 staining with the PIP2 sensor PLCg-PH- GFP at the equatorial wedge and at the site of cell-cell contacts in wild-type nephrocytes. Both sites contain two closely apposed membranes but as shown in Fig. 3A,B, signal intensity at the equator is double than that at regions of cell contacts. In addition, we have also included similar quantifications in *pyd*^{ex147} mutant nephrocytes, where equatorial membrane ingressions do not form, showing that PIP2 still accumulates twice at the equatorial cortex than at cell-cell contacts (Figs 3B, 4A,C-C’).

Minor points in the text:

1. Missing word “on” after “confer” (line 59).

Authors’ response:
We thank the reviewer for pointing out this typographical error that we have changed in the revised version (line 53).

2. Define sns-GCN-nGFP when first mentioned (line 164).

Authors’ response:
Following the reviewer suggestion we have added “, expressing GFP in the nuclei of nephrocytes,” after the first mention of sns-GCN-nGFP (line 165).

© 2023. Published by The Company of Biologists under the terms of the Creative Commons Attribution License (https://creativecommons.org/licenses/by/4.0/).

3. "Effectively" (line 176) is unclear and could be deleted, and the sentence (esp. line 177) should be rewritten for clarity.

Authors' response:
According to the reviewer's suggestion, we have rewritten the sentence as follows: "Indeed, embryos deficient for the slit diaphragm proteins *duf* (*Df(1)*w^{67k30}, also removing *duf* paralogue *roughest*), and *sns* (*sns*^{XB3}), both presenting a complete block of myoblast fusion (Bour et al., 2000; Ruiz-Gómez et al., 2000 and Fig. S1A,B,C, compare with Fig. S1D,E), exhibit binucleated garland nephrocytes (Fig. S1B',C', see also Weavers et al., 2009)" (starting line 176).

4. Similarly, at the bottom of the page, it would help to start new sentence with "In contrast," instead of "whereas" (line 180) for clarity.

Authors' response:
We have taken into account this suggestion and modified the text accordingly (line 179).

5. "by large" is confusing (lines 218-219) and could be deleted.

Authors' response:
We have taken into account this suggestion and modified the text accordingly (line 216).

6. "Noteworthy" (line 273) could be replaced by "Of note,".

Authors' response:
We have taken into account this suggestion and modified the text accordingly (line 273).

7. For clarity, move "PIP2" (line 277) to the next line, before "would".

Authors' response:
We have taken into account this suggestion and modified the text accordingly (line 276).

8. **Fix typos: "slit" (line 343) and "wild-type" (line 369).**

Authors' response:
We have taken into account this suggestion and modified the text accordingly.

Minor comments on the figures:

1. Colored labels on the gray boxes above the panels (Figs 1, 3, 4, S1, S3) are difficult to read, especially when printed out.

Authors' response:
We have modified the colour of the boxes to make them easier to read.

2. In general, it would help to show single color images (Fig. 1B', F''', G'', I) in grayscale rather than color, as grayscale is easier to see on a black background. Also, for two color images, magenta and green is better than red and green for colorblind readers.

Authors' response:
We thank the reviewer very much for these comments and have changed single colour images to greyscale and red for magenta in all images displaying double staining.

3. Some of the arrowheads are extremely small and difficult to see when printed (for example, in Fig. 1B, B'). Similarly, some of the arrows are extremely skinny and hard to see (Fig. 1C-C'', D-D', E, K', L'). The blue asterisk (Fig. 1L', upper inset) is nearly impossible to see.

© 2023. Published by The Company of Biologists under the terms of the Creative Commons Attribution License (https://creativecommons.org/licenses/by/4.0/).

<u>Authors' response:</u>
We apologise for this, and have increased the size of the symbols as suggested.

<u>Minor comments on the supplementary figure legends:</u>

1. The interpretation in the last sentence of the Fig. S1 legend, "indicating that binucleation of nephrocytes is the result of cell divisions" (lines 15-16) seems an overstatement; suggest replacing "is the result of" with "correlates with".

<u>Authors' response:</u>
We agree and have followed the reviewer's suggestion.

2. It's unclear what is meant by "just opposite to the outer membrane" (line 36) in the Fig. S2 legend.

<u>Authors' response:</u>
To avoid confusion we have removed this sentence from the revised legend.

Reviewer #3
<u>Major points</u>:

Query 1. Fig. 1A: Why are Pyd and Duf not clustered at all tricellular junctions? Some spots are clearly outside tricellular junctions, although it is hard to judge in this resolution. Thus, a co-stainining with a marker of tricellular junctions (sidekick) would help here - staining of sidekick (Fig. 1B) alone is dispensable in this context.

<u>Authors' response:</u>
We thank the reviewer for this comment. After performing the double staining for Pyd and Sdk suggested by the reviewer, we found that both proteins clearly colocalise, as shown in the revised Fig. 1B-B". Note however, that nephrocytes are mesodermal cells that lack canonical tricellular junctions, and this might be the reason why the accumulation of slit diaphragm proteins and Sdk extends beyond the site of tricellular contacts.

Query 2. Fig. 3: Intensities of PLC-PH-GFP accumulation at cytokinesis furrows in contrast to other membranes should be quantified.

<u>Authors' response:</u>
We thank all reviewers for pointing this out and understand the concern raised by them regarding PIP2 enrichment in the equatorial membrane. To clarify this question, we are presenting in the revised version of the manuscript additional data that substantially support our conclusion that such PIP2 enrichment is not due to the presence of the double layer of plasma membrane. We have quantified the intensity of PIP2 staining with the PIP2 sensor PLCg-PH- GFP at the equatorial wedge and at the site of cell-cell contacts in wild-type nephrocytes. Both sites contain two closely apposed membranes but as shown in Fig. 3A,B, signal intensity at the equator is double than that at regions of cell contacts. In addition, we have also included similar quantifications in pyd^{ex147} mutant nephrocytes, where equatorial membrane ingressions do not form, showing that PIP2 still accumulates twice at the equatorial cortex than at cell-cell contacts (Figs 3B, 4A,C-C').

Query 3. A slit diaphragm marker (Pyd/Duf) as well as stainings against the Septin Peanut should be used together with PLC-PH-GFP to verify that slit diaphragm proteins accumulate at sites with high PIP2 density at the cytokinesis ring.

<u>Authors' response:</u>
We agree with the reviewer that such a triple staining would be ideal. But unfortunately, the different fixation protocols required for slit diaphragm protein staining (heat-fixation) and GFP (PFA fixation), prevented us to perform the suggested triple staining. However, we performed double staining to reveal Peanut accumulation in PIP2-enriched domains as shown in the revised Fig. S3C-C". And we have also shown the co-localisation of PIP2 with the HRP epitope (Figs 3A, 4C) and that of the HRP epitope with slit diaphragm proteins in Fig. S3A. We think that

© 2023. Published by The Company of Biologists under the terms of the Creative Commons Attribution License (https://creativecommons.org/licenses/by/4.0/).

together these data prove the accumulation of slit diaphragm proteins at sites with high PIP2 density at the cytokinetic ring.

Query 4. Fig. 3D: DAPI staining (cp Fig. 3B''') would help to identify binucleated nephrocytes.

Authors' response:

The nephrocytes shown in Fig. 3D (3H-H'" in the revised Fig. 3) were counterstained with DAPI to reveal the nuclei. However in the Z projection shown in Fig. 3H DAPI staining was omitted because it was blurred out by Zipper accumulation. We have now included a novel supplementary figure showing a stack of several sections (the same string of nephrocytes on Fig. 3H) of nephrocytes stained for Zipper and Duf where DAPI staining is included (Fig. S4). Note in A how Zipper accumulation mask the position of some of the nuclei. To establish the localisation of Zipper and Duf in relation to the nuclei, split channels are shown in Fig. S4A',A".

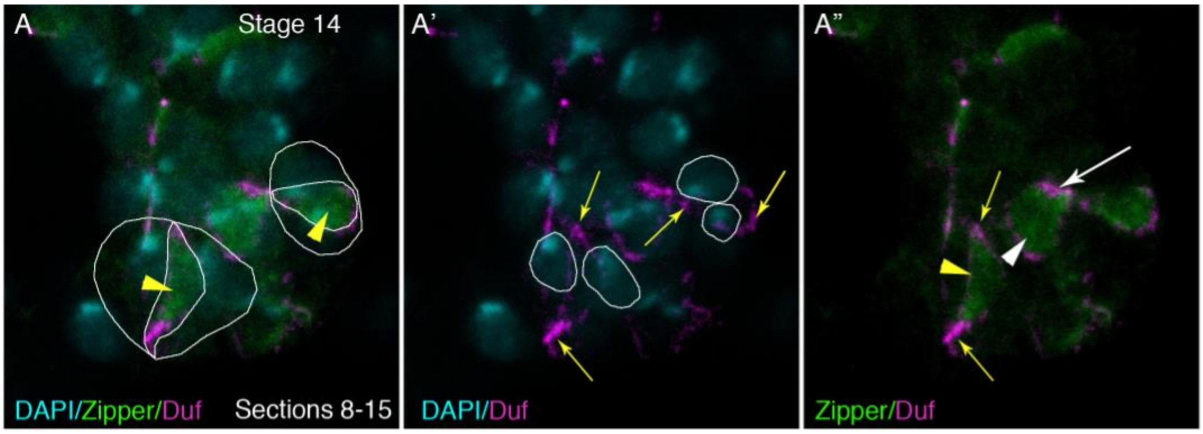

Fig. S4 (related to Fig. 3H). Accumulation of Zipper and Duf in stage 14 wild-type garland nephrocytes. (A-A") Detail of a Z projection of sections 8 to 15 of the string of nephrocytes from Fig. 3H, to show the accumulation of Zipper and Duf in relation to the position of the sibling nuclei. (A) Cell contours and equatorial wedges (accumulating Zipper, arrowheads) are outlined for two cells. (A') The pairs of sibling nuclei of these cells are highlighted; arrows point to Duf accumulation at the equatorial cortexes. (A") Equatorial wedges and cortexes are marked by arrowheads and arrows, respectively. White symbols point to the cell similarly marked in Figs 3H-H".

Query 5. Fig. 4: Are slit diaphragms recovered at later stages (e.g. in larval stages), if acytokinetic cell division is blocked (maybe here a guide or shRNA line against CycA instead of cycA mutants must be used)?

Authors' response:
Unfortunately the suggested experiment cannot be performed since RNAi-mediated attenuation is not efficient during embryogenesis and zygotic *cycA* mutants are embryonic lethal. We have however shown that when we force cytokinesis to occur in the extra post-blastoderm mitosis in garland nephrocytes, the density of slit diaphragms in larval stages is similar to that of wild-type nephrocytes (see novel Fig. 5C,C'). This indicates that despite the delay on the onset of slit diaphragm assembly (Fig. 5E-F'"), once they start to form, they continue to do so until covering the entire surface of the cell. Note in addition, that in larval pericardial nephrocytes, which do not have an extra post-blastoderm division, and therefore do not assemble slit diaphragms during embryogenesis, the density of slit diaphragms is similar to that of garland nephrocytes. This allows us to predict that the same would occur in garland nephrocytes if we could have prevented the extra post-blastoderm mitosis.

Query 6. Fig. 4J: The number of slit diaphragms formed in Pebble/Sqh-overexpressing nephrocytes should be quantified to demonstrate that nephrocytes, which complete cytokinesis exhibit no defects in slit diaphragm formation in larval stages.

© 2023. Published by The Company of Biologists under the terms of the Creative Commons Attribution License (https://creativecommons.org/licenses/by/4.0/).

Authors' response:
We thank the reviewer for this constructive suggestion, and accordingly, we have included both quantifications in the novel Fig. 5C,C'.

Query 7. Fig. 4K: The control situation of nephrocytes at stage 16 should be shown (as in Fig. 1E) for better comparison).

Authors' response:
Following the reviewer's suggestion, the requested control is included in Fig. 5D,D'.

Query 8. Overexpression of Pbl in otherwise wild type nephrocytes is capable to induce a completion of cytokinesis with low penetrance. Why is the accumulation of slit diaphragms incomplete/delayed in all cells (mono- and binucleated cells) at stage 16, which hints at a role of Pbl/Myosin contraction in positioning/stabilizing slit diaphragms independently of the cleavage furrow?

Authors' response:
As was indicated in the main text and in the legend to Fig. 4 of the previous version of the manuscript, the delay in slit diaphragm assembly was only observed in mononucleated nephrocytes (cells 1, 2 and 3 in previous Fig. 4K-K"), whereas binucleated cells presented the characteristic distribution of slit diaphragm proteins at stage 16 (cell 4 in Fig. 4K', white arrow). In the revised version of the manuscript we have added an additional panel displaying single channel for Pyd in grey to clarify this point (novel Fig. 5E-E'").

Query 9. Line 350ff: In my eyes, the explanation of the recruitment of additional slit diaphragms is not logic: The first strand of slit diaphragms assemble at the cleavage furrow due to incomplete cytokinesis, which was nicely demonstrated - how should these complexes recruit the next strand, approximately 300-400nm apart from the first strand?

Authors' response:
We apologise if in the previous version of the manuscript the proposed model to account for additional slit diaphragm formation wasn't clearly explained. Our hypothesis "that the first slit diaphragms formed at the equatorial cortex would also help maintain membrane heterogeneity by acting as a nucleation centre for the recruitment of additional slit diaphragm complexes, until eventually, the entire nephrocyte outer membrane will be covered with slit diaphragms" was based in the observations of the evolution of slit diaphragm proteins distributions from stage 14 to 15. We described that their distribution in an equatorial-located ring at stage 14, became "distorted at stage 15, when additional slit diaphragm complexes accumulate at locations close to the initial positions (Fig. 1D-D", arrows)". To clarify this point we have rephrased this paragraph as follows: "At stage 15, this sharp ring becomes distorted, presenting protrusions extending form it, when additional slit diaphragm complexes accumulate at locations close to their pre-existing positions (Fig. 1D-D", arrows)". We still put forward this hypothesis, which we think is also supported by the observation of the pattern of slit diaphragm recovery we previously reported in Carrasco-Rando et al., 2019. There, we described the temporal sequence of slit diaphragm formation in *pyd* mutants after delivery of exogenous Pyd protein. In this scenario, the first slit diaphragms appeared close to cell-cell contacts, and the next, close to the previous ones. We interpret that the accumulation of slit diaphragms proteins in PIP2-enriched domains somehow facilitates the expansion of these domains in the plasma membrane, by an hitherto unknown mechanism, favouring the recruitment of additional slit diaphragm components.

We understand the concern raised by the reviewer regarding "– how should these complexes recruit the next strand, approximately 300-400nm apart from the first strand?". If the reviewer refers to the stage 16 TEM in Fig. 1L,L', showing a channel quite away from the equatorial region, we would like to stress that this corresponds to a single section, and a 3D reconstruction will be required to visualise the whole distribution of slit diaphragms (that now are distributed throughout the entire cell surface).

Query 10. Fig. 4L: I suggest to take out the data of apical polarity regulators (aPKC) affecting early slit diaphragm formation, otherwise more experiments are needed to address this

© 2023. Published by The Company of Biologists under the terms of the Creative Commons Attribution License (https://creativecommons.org/licenses/by/4.0/).

(interesting) question, e.g. by downregulation/mutation of aPKC and Baz (which directly binds to PI(4,5)P2). In the current version, the results that overexpression of an activated version of aPKC delays acytokinetic division and slit diaphragm formation remains unexplained (or is explained by functions of aPKC which are independent of its role in polarity) and does not contribute to the main findings of the manuscript.

Authors' response:
In this study, we investigated whether aPKC plays an instructive role providing positional information for slit diaphragm assembly in nephrocytes. Our data suggest that this is not the case, since overexpression of constitutively-active $aPKC^{DN}$, homogeneously distributed in the membrane, nuclei and cytoplasm of nephrocytes, does not alter the localisation of slit diaphragm proteins, which continue to accumulate in the equatorial cortex of the nephrocyte,
thus pointing to PIP2 as the main determinant of slit diaphragm assembly sites. Accordingly, we decided to maintain these data in the revised manuscript.

Query 11. Finally, in the title the authors suggest a causative role for PI(4,5)P2 leading to slit diaphragm assembly. While this is quite likely and suggested by another study (Gass et al. 2022), too, the shown experiments do not (yet) support this conclusion. Downregulation of PI(4,5)P2 in developing nephrocytes should block accumulation of slit diaphragm components at the cytokinesis furrow and demonstrate a causative role of this phospholipid as suggested by the authors.

Authors' response:
We agree that studying the effect of PIP2 downregulation would be ideal, but as explained in response to a similar query of reviewer 1, experimental approaches to decrease PIP2 levels in embryonic nephrocytes are not feasible due to technical limitations as now indicated in the text. Thus, optogenetic manipulation of PIP2 levels in embryonic nephrocytes is precluded by their location deep inside the embryo and floating in the haemolymph. We considered to modify PIP2 levels by manipulating the Inositol 5-phosphatase dOCRL, which desphosphorylates PIP2.

However, analysis of the effect of dOCRL loss in nephrocytes was prevented by the persistent maternal activity in null $docrl^{\Delta 3}$ mutants, which survive until larval or pupal stages. In addition, RNAi-mediated attenuation is not possible, since it doesn't work efficiently during embryogenesis and in the case of *docrl*, the available RNAi lines have been reported to be insufficient to deplete *docrl*. Hence, we resorted to increase PIP2 levels in nephrocytes by overexpressing UAS-*docrl* with *VT016847-Gal4*. This experimental approach led to a premature accumulation of slit diaphragm proteins in the outer nephrocyte membrane, thus suggesting that PIP2 membrane enrichment directly affects the stabilisation of the diaphragm components in flies. These results are presented in the new Fig. 4D-D".

Minor points
1. Fig. 1C – A DAPI staining labelling the nuclei (as shown in C'') would help to distinguish single binucleated nephrocytes.

Authors' response:
Please note that panels in Fig. 1C'-C" correspond to the area boxed in Fig. 1C. Nuclei are omitted in Fig. 1C to facilitate visualisation of Duf and Pyd accumulation in the equatorial ring.

2. Fig. 2: Quantifcation of nephrocyte cell types should be displayed in the main figure, not in the supplements.

Authors' response:
Following the reviewer's suggestion the quantification is included in revised Fig. 2B.

3. Line 329: It should become clear, that "experimental nephrocytes" are nephrocytes with an overexpression of Pbl.

Authors' response:
We have modified the main text according to this suggestion (line 342).

© 2023. Published by The Company of Biologists under the terms of the Creative Commons Attribution License (https://creativecommons.org/licenses/by/4.0/).

4. Line 369: Typo: wild-type
5. Line 343: Typo: slit diphragms

Authors' response:
We thank the reviewer for pointing out these typographical errors that we have corrected in the revised version.

Second decision letter

MS ID#: DEVELOP/2023/201708

MS TITLE: An acytokinetic cell division creates PIP2-enriched membrane asymmetries leading to slit diaphragm assembly in Drosophila nephrocytes

AUTHORS: Marta Carrasco-Rando, Joaquim Culi, Sonsoles Campuzano, and Mar Ruiz-Gómez
ARTICLE TYPE: Research Article

I am happy to tell you that your manuscript has been accepted for publication in Development, pending our standard ethics checks.

Reviewer 1

Advance summary and potential significance to field

See past summary.

Comments for the author

The authors have effectively addressed my past concerns with new data and revisions to the text. The manuscript also carefully discusses experimental limitations.
Overall, it is a carefully conducted and substantial study that advances the understanding of how Drosophila embryo nephrocytes develop their unique cell structure for hemolymph filtration.

Reviewer 3

Advance summary and potential significance to field

In their manuscript, Carrasco-Rando et al. describe the process of slit diaphragm formation in the development of Drosophila nephrocytes, cells, which show similarity with mammalian podocytes. The authors convincingly show that incomplete cytokinesis generates the first membrane asymmetry with local accumulation of PI(4,5)P2 and slit diaphragm components at this site. Forced completion of cytokinesis delays the formation of slit diaphragms. This manuscript substantially enhances our understanding of how membrane invaginations and slit diaphragms in nephrocytes are formed.

Comments for the author

The authors fully addressed all my questions and I am happy to recommend this nice paper for publication.

© 2023. Published by The Company of Biologists under the terms of the Creative Commons Attribution License (https://creativecommons.org/licenses/by/4.0/).
